# Phenotype, serotype, and data-driven clustering reveal complementary dimensions of heterogeneity in ANCA-associated vasculitis: a multicentre Japanese cohort (J-CANVAS)

**DOI:** 10.1007/s00296-025-06014-y

**Published:** 2025-11-12

**Authors:** Genki Kidoguchi, Yusuke Yoshida, Satoshi Omura, Daiki Nakagomi, Yoshiyuki Abe, Makoto Wada, Naoho Takizawa, Atsushi Nomura, Yuji Kukida, Naoya Kondo, Hirosuke Takagi, Koji Endo, Naoto Azuma, Tohru Takeuchi, Shoichi Fukui, Kazuro Kamada, Ryo Yanai, Yusuke Matsuo, Yasuhiro Shimojima, Ryo Nishioka, Ryota Okazaki, Tomoaki Takata, Mayuko Moriyama, Ayuko Takatani, Yoshia Miyawaki, Tsuyoshi Shirai, Hiroaki Dobashi, Takafumi Ito, Isao Matsumoto, Toshihiko Takada, Yutaka Kawahito, Toshiko Ito-Ihara, Takashi Kida, Nobuyuki Yajima, Takashi Kawaguchi, Shintaro Hirata

**Affiliations:** 1 Department of Clinical Immunology and Rheumatology, Hiroshima University Hospital, 1-2- 3 Kasumi, Minami-ku, Hiroshima, 734-8551 Japan; 2 Inflammation and Immunology, Graduate School of Medical Science, Kyoto Prefectural University of Medicine, Kyoto, Japan; 3 Department of Rheumatology, University of Yamanashi Hospital, Yamanashi, Japan; 4 Department of Internal Medicine and Rheumatology, Juntendo University, Tokyo, Japan; 5 Center for Rheumatic Disease, Japanese Red Cross Society Kyoto Daiichi Hospital, Kyoto, Japan; 6 Department of Rheumatology, Chubu Rosai Hospital, Nagoya, Aichi Japan; 7 Immuno-Rheumatology Center, St Luke’s International Hospital, Tokyo, Japan; 8 Department of Rheumatology, Japanese Red Cross Society Kyoto Daini Hospital, Kyoto, Japan; 9 Department of Nephrology, Kyoto Katsura Hospital, Kyoto, Japan; 10 Department of Hematology and Rheumatology, Kagoshima University Hospital, Kagoshima, Japan; 11 Department of General Internal Medicine, Tottori Red Cross Hospital, Tottori, Japan; 12 Department of Diabetes, Endocrinology and Clinical Immunology, Hyogo Medical University School of Medicine, Hyogo, Japan; 13 Department of Internal Medicine (IV), Osaka Medical and Pharmaceutical University, Osaka, Japan; 14 Department of Immunology and Rheumatology, Division of Advanced Preventive Medical Sciences, Nagasaki University Graduate School of Biomedical Sciences, Nagasaki, Japan; 15 Department of Rheumatology, Endocrinology and Nephrology, Faculty of Medicine, Graduate School of Medicine, Hokkaido University, Sapporo, Hokkaido Japan; 16 Division of Rheumatology, Department of Medicine, Showa Medical University School of Medicine, Tokyo, Japan; 17 Department of Rheumatology, Tokyo Kyosai Hospital, Tokyo, Japan; 18 Department of Rheumatology, Graduate School of Medical and Dental Sciences, Institute of Science Tokyo (formerly Tokyo Medical and Dental University, Bunkyo-ku, Japan; 19 Department of Medicine (Neurology and Rheumatology), Shinshu University School of Medicine, Nagano, Japan; 20 Department of Rheumatology, Graduate School of Medical Science, Kanazawa University, Kanazawa, Japan; 21 Division of Respiratory Medicine and Rheumatology, Department of Multidisciplinary Internal Medicine, Faculty of Medicine, Tottori University, Tottori, Japan; 22 Division of Gastroenterology and Nephrology, Tottori University, Tottori, Japan; 23 Department of Rheumatology, Shimane University Faculty of Medicine, Shimane, Japan; 24 Rheumatic Disease Center, Sasebo Chuo Hospital, Nagasaki, Japan; 25 Department of Public Health, Nagasaki University Graduate School of Biomedical Sciences, Nagasaki, Japan; 26 Department of Nephrology, Rheumatology, Endocrinology and Metabolism, Dentistry and Pharmaceutical Sciences, Okayama University Graduate School of Medicine, Okayama, Japan; 27 Department of Rheumatology, Tohoku University Hospital, Sendai, Miyagi Japan; 28 Division of Hematology, Rheumatology and Respiratory Medicine, Department of Internal Medicine, Faculty of Medicine, Kagawa University, Kagawa, Japan; 29 Division of Nephrology, Department of Internal Medicine, Teikyo University Chiba Medical Center, Chiba, Japan; 30 Department of Rheumatology, Institute of Medicine, University of Tsukuba, Tsukuba, Ibaraki Japan; 31 Department of General Medicine, Shirakawa Satellite for Teaching and Research (STAR), Fukushima Medical University, Shirakawa, Fukushima Japan; 32 The Clinical and Translational Research Center, University Hospital, Kyoto Prefectural University of Medicine, Kyoto, Japan; 33 Department of Clinical Assessment, Tokyo University of Pharmacy and Life Sciences, Tokyo, Japan

**Keywords:** Anti-Neutrophil cytoplasmic Antibody-Associated vasculitis, Classification, Phenotype, Serotyping, Cluster analysis

## Abstract

**Objective:**

To compare clinicopathological phenotype-based, anti-neutrophil cytoplasmic antibody (ANCA) serotype-based, and unsupervised data-driven classifications in relation to clinical outcomes and patient heterogeneity in a large Japanese cohort with ANCA-associated vasculitis (AAV).

**Methods:**

This multicentre, retrospective cohort study analysed data from a nationwide Japanese registry of 729 newly diagnosed patients with granulomatosis with polyangiitis (GPA) or microscopic polyangiitis (MPA), all positive for myeloperoxidase (MPO)- or proteinase 3 (PR3)-ANCA. Patients were classified by phenotype, serotype, combined phenotype-serotype groupings, and data-driven clustering based on baseline clinical and laboratory features. Associations with clinical outcomes—including mortality, relapse, and response to rituximab (RTX) versus cyclophosphamide (IVCY)—were evaluated using inverse probability of treatment weighting (IPW).

**Results:**

Phenotype-based classification more accurately distinguished all-cause mortality risk (MPA vs. GPA: hazard ratio [HR] 2.53, 95% CI 1.34–4.76). Combined phenotype-serotype analysis identified MPO-MPA patients with the highest mortality (HR 3.45, 95% CI 1.09-11.0, vs. PR3-GPA) and PR3-GPA with the highest severe relapse. Discordant groups, such as MPO-GPA, demonstrated unique clinical characteristics. After IPW adjustment, no significant difference in 24-week remission rates was observed between RTX and IVCY across classifications, both overall (RR 1.02, 95% CI 0.95–1.09) and within subgroups. Unsupervised clustering identified four distinct clinical subgroups, with limited concordance with conventional phenotype or serotype classifications.

**Conclusion:**

Phenotype and serotype classifications provide complementary, not competing, prognostic insights in Japanese patients with AAV. Data-driven clustering revealed additional clinical heterogeneity not captured by traditional systems, underscoring the need for integrated, multi-dimensional stratification approaches to improve personalised risk assessment and treatment strategies.

**Supplementary Information:**

The online version contains supplementary material available at 10.1007/s00296-025-06014-y.

## Introduction

Anti-neutrophil cytoplasmic antibody (ANCA)-associated vasculitis (AAV) is a systemic autoimmune condition characterised by inflammation of small vessels, primarily driven by ANCAs targeting proteinase 3 (PR3) or myeloperoxidase (MPO). Historically, AAV subtypes, such as granulomatosis with polyangiitis (GPA) and microscopic polyangiitis (MPA), have been distinguished based on clinicopathological findings [1]. However, classification based on the ANCA serotype (PR3-AAV vs. MPO-AAV) has gained increasing prominence for several reasons. This approach is closely linked to underlying genetic variations, enables faster and more clinically relevant diagnoses, and provides independent prognostic insights into relapse risk, and shows strong concordance with the latest classification criteria [2–5].

The optimal method for classifying AAV remains debated, largely because current systems, based solely on clinicopathological features or ANCA serotypes, may not fully capture patient heterogeneity [6, 7]. While clinicopathological grouping has been reported to be a strong prognostic indicator, significant overlap exists, as illustrated by cases such as MPO-ANCA-positive GPA. Additionally, patient outcomes vary widely within traditional categories; for example, overall survival and relapse-free survival in GPA differ according to ANCA status (PR3-positive, MPO-positive, or negative) [8]. This complexity suggests that relying solely on dichotomous classifications is inadequate and highlights the need for a more integrated framework to better reflect the full AAV spectrum.

Given the limitations of current AAV classification systems and the ongoing debate surrounding them, comparative evaluations of their prognostic value are warranted. Therefore, we aimed to assess the predictive value of traditional phenotype-based classification (GPA/MPA), ANCA serotype (PR3/MPO), and data-driven clustering approaches in a large Japanese AAV cohort. Specifically, we compared these classification methods in relation to key clinical outcomes, including all-cause mortality, renal outcomes, severe relapse, serious infection, and remission rates following induction therapy, using retrospective data analysis that incorporated survival modelling and cluster analysis.

## Materials and methods

### Study design and setting

We conducted a multicentre, retrospective cohort study using data from the Japan Collaborative Registry of ANCA-Associated Vasculitis (J-CANVAS). J-CANVAS is an ongoing multicentre registry in Japan that has enrolled patients with AAV from 30 participating institutions (Online Resource 1, Supplementary Table 1). For this analysis, we included patients enrolled between 1 January 2017 and 31 March 2023. Clinical data for these patients were collected and followed up until 31 March 2024. This study was reported in accordance with the Strengthening the Reporting of Observational Studies in Epidemiology guidelines.

### Participants

Patients were eligible for inclusion if they were aged 20 years or older at the time of AAV diagnosis (for newly diagnosed cases) or at the time of relapse (for relapsing cases). This age threshold was chosen to focus on an adult population, as paediatric-onset AAV can present differently from adult-onset disease [9]. Inclusion criteria required a documented diagnosis of either GPA or MPA, along with positive serology for either MPO-ANCA or PR3-ANCA. Patients diagnosed with eosinophilic granulomatosis with polyangiitis (EGPA) or those who were negative for both MPO-ANCA and PR3-ANCA were excluded. Patients who tested positive for both MPO-ANCA and PR3-ANCA were assigned to either the MPO-ANCA or PR3-ANCA groups based on the higher antibody titre. If titres were comparable or unavailable, classification was determined by the treating physician based on clinical relevance. The primary analysis focused on patients with newly diagnosed AAV to minimise potential confounding factors from prior treatments or prolonged disease duration. A separate cohort of patients experiencing severe relapse, defined as organ-threatening or life-threatening disease according to the European League Against Rheumatism recommendations [10], and meeting other eligibility criteria, was used for sensitivity analyses.

### Data collection and variables

#### Baseline characteristics

We collected demographic data at the time of diagnosis or severe relapse, including age, sex, and comorbidities. Laboratory parameters included serum creatinine level, estimated glomerular filtration rate (eGFR) calculated using the Japanese equation, C-reactive protein (CRP), and Krebs von den Lungen-6 (KL-6) levels. Baseline immunosuppressive treatments administered prior to diagnosis were also recorded. Organ involvement was categorised into the following systems: constitutional, musculoskeletal, skin, mucosa, eyes, ear/nose/throat (ENT), pulmonary, cardiovascular, gastrointestinal, renal, and central and peripheral nervous systems. This detailed, system-based assessment, consistent with previous AAV research [11], enabled a granular characterisation of clinical phenotypes across different AAV classifications. It also provided comprehensive input for subsequent data-driven clustering analysis, complementing overall disease activity measured by the Birmingham Vasculitis Activity Score (BVAS) version 3.0 [12]. All assessments were conducted by physicians experienced in vasculitis management at each participating centre.

### Patient classification

Patients were classified using two primary systems:

- *Phenotype-based classification*: Patients were categorised as having either GPA or MPA based on the 2012 Chapel Hill Consensus Conference nomenclature and the European Medicines Agency algorithm [1, 13].
- *Serotype-based classification*: Patients were grouped according to their ANCA specificity, either PR3-ANCA or MPO-ANCA positivity. This combined classification approach yielded four subgroups: MPO-MPA, PR3-MPA, MPO-GPA, and PR3-GPA. ANCA testing was conducted at each participating centre using enzyme-linked immunosorbent assay, following local protocols [14].

### Outcomes

The outcomes of interest included all-cause mortality, defined as death from any cause during the follow-up period; a composite renal outcome, comprising end-stage kidney disease, initiation of renal replacement therapy, or a sustained ≥ 50% decline in eGFR from baseline, lasting at least 3 months; severe relapse, characterised as either a major relapse or an organ- or life-threatening disease, as defined by the 2022 AAV recommendations; and serious infection, defined as infection requiring hospitalisation or intravenous antimicrobial therapy [10]. Treatment response was assessed by evaluating remission status at week 24 following the initiation of induction therapy. Remission was defined as a BVAS of 0, regardless of the continued use of immunosuppressive drugs.

### Statistical analysis

#### Descriptive statistics and survival analysis

Baseline characteristics were summarised as counts and percentages for categorical variables and medians with interquartile ranges for continuous variables. Comparisons between groups were performed using the chi-square or Fisher’s exact tests for categorical variables and the Mann–Whitney U test for continuous variables. Overall survival (OS), defined as the time from diagnosis to death from any cause, and relapse-free survival (RFS), defined as the time from diagnosis to either disease-related death or severe relapse, were estimated using the Kaplan–Meier method. Differences between groups were assessed using the log-rank test. The cumulative incidence of severe relapse, with death considered a competing risk, was estimated using the Fine-Gray model and compared between groups using Gray’s test. Crude hazard ratios (HRs) for OS and RFS were obtained from Cox proportional hazards models, while crude subdistribution hazard ratios for severe relapse were derived from Fine-Gray models, each with corresponding 95% confidence intervals (CIs) [15]. A two-sided p-value of < 0.05 was considered statistically significant.

#### Treatment response analysis

Treatment response was defined as remission, indicated by a BVAS score of 0, at 24 weeks after induction therapy with either rituximab (RTX) or intravenous cyclophosphamide (IVCY). Missing outcome data and key covariates (serum creatinine and albumin), (summarized in Online Resource 1, Supplementary Table 2), were addressed using multiple imputations by chained equations (MICE), generating 50 imputed datasets. The imputation models included the outcome, treatment assignment, propensity score covariates, and subgroup variables. To adjust for confounding and estimate the average treatment effect, inverse probability of treatment weighting (IPW) based on propensity scores (PS) was applied [16]. PS was estimated using logistic regression, with baseline covariates selected based on directed acyclic graphs (Online Resource 1, Supplementary Data and Supplementary Fig. 1) [17, 18]. Covariate balance after weighting was assessed using standardised mean differences, with values below 0.1 indicating acceptable balance [19]. The overall adjusted risk ratio (RR) and 95% CI for 24-week remission (IVCY vs. RTX) were estimated using weighted modified Poisson regression with a log link function [20]. Results from the 50 imputed datasets were combined using Rubin’s rules. Effect modification was assessed using stratified analyses by phenotype, serotype, and combined subgroups. Subgroup-specific RRs and 95% CIs were estimated using weighted models incorporating treatment-by-subgroup interaction terms. Interaction p-values were used to evaluate effect modification by phenotype and serotype. Technical limitations precluded an overall interaction test for the combined subgroups.

#### Data-driven clustering analysis

Missing baseline clustering variables (e.g., serum creatinine) were handled using MICE, generating 10 imputed datasets. Within each dataset, unsupervised model-based clustering using Gaussian mixture models was applied, incorporating baseline characteristics, organ involvement patterns, and laboratory markers (the complete list of variables is provided in Online Resource 1, Supplementary Data). Input variables included age, BVAS, creatinine, CRP, and organ involvement patterns. Pulmonary involvement was further subdivided into nodules/cavities and interstitial lung disease (ILD), as these manifestations may reflect distinct pathophysiological processes in AAV [21, 22]. Serotype was initially excluded from clustering to avoid circular reasoning when compared with conventional classifications. All input variables were standardised before analysis. The optimal cluster number (tested from 1 to 5) was selected based on the Bayesian Information Criterion, with lower values indicating better model fit. Final cluster membership for each patient was determined by their most frequent (modal) assignment across the 10 imputed datasets. The resulting clusters were characterised and visualised using radar charts. Cluster structure from a representative imputed dataset was further visualised using the mclustDR R package [22]. Concordance between the data-driven clusters and traditional phenotypes or serotypes was evaluated using heatmaps.

#### Sensitivity analyses

Three main sensitivity analyses were conducted. First, the primary comparative analyses were repeated using the full cohort, which included both newly diagnosed and relapsed patients (*n* = 874). Second, the robustness of the treatment effect estimation was assessed using complete case analysis (CCA). This involved re-estimating the IPW effect within the CCA subset and additionally applying targeted maximum likelihood estimation (TMLE) to provide an alternative estimate of the treatment effect on the RR scale for comparison [23]. Third, the model-based clustering analysis was repeated with the ANCA serotype included as an input variable. All statistical analyses were performed using R version 4.4.2 (R Foundation for Statistical Computing, Vienna, Austria). During the statistical analysis, the large language model Gemini 2.5 Pro was employed to assist in troubleshooting the code. All AI-generated suggestions were reviewed and implemented by the authors, who retain full responsibility for the final analysis and the content of this work.

## Results

### Study population

A total of 1,107 eligible patients with MPA or GPA were initially identified from the J-CANVAS registry during the study period. After excluding 214 patients with EGPA and 19 patients negative for both MPO-ANCA and PR3-ANCA, 874 patients remained eligible. Of these, 729 (83.4%) were newly diagnosed cases, and 145 (16.6%) were relapsed cases. The participant selection process is illustrated in Online Resource 1, Supplementary Fig. 2.

### Comparison between phenotype-based and serotype-based classifications

Baseline characteristics and crude outcomes for the newly diagnosed cohort (*n* = 729), stratified by phenotype (MPA vs. GPA) and serotype (MPO-ANCA vs. PR3-ANCA), are presented in Table 1. Both classification systems revealed significant differences in patient age between subgroups (MPA > GPA, *p* < 0.001; MPO > PR3, *p* < 0.001). However, other baseline variables, such as sex distribution, disease activity (BVAS), and CRP levels, were similar between subgroups within each classification system. Phenotype and serotype classifications were strongly associated, although some discordance was observed; for example, 16.8% of MPO-ANCA-positive patients had GPA, and 20.3% of PR3-ANCA-positive patients had MPA.

The ability to distinguish patients based on organ involvement patterns varied between the classification methods. For instance, significant differences in the frequencies of musculoskeletal, lung, and central nervous system involvement were observed only when comparing phenotypes (MPA vs. GPA). Conversely, significant differences in constitutional symptoms and mucosal involvement were observed only when comparing serotypes (MPO vs. PR3). Both classification approaches revealed significant differences in the frequencies of eye and kidney involvement between their respective subgroups (Table 1). Similarly, baseline serum creatinine and KL-6 levels differed significantly within both classification systems (Table 1).

**Fig. 1:**
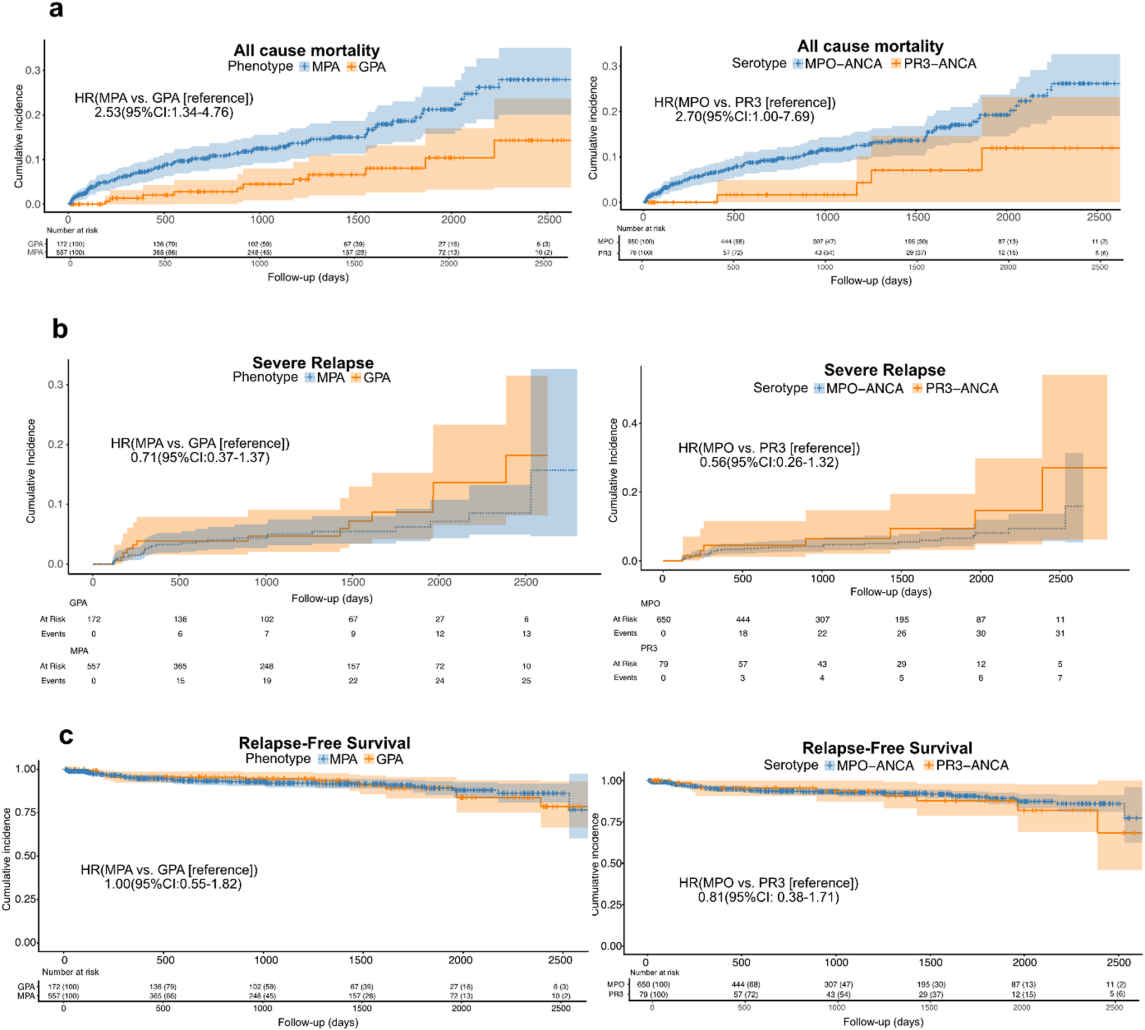
Survival and relapse outcomes according to phenotype and serotype in newly diagnosed AAV. (a) All-cause mortality (KM curves), (b) Severe relapse (CIF curves accounting for death as a competing risk), (c) Relapse-free survival (KM curves; event defined as death or severe relapse). The left panels show stratification by phenotype (MPA vs. GPA [reference]), while the right panels show stratification by serotype (MPO-ANCA vs. PR3-ANCA [reference]). Crude HRs with 95% CIs and numbers at risk are presented

Regarding clinical outcomes in unadjusted analyses (Table 1; Fig. 1), phenotype classification better discriminated the risk of all-cause mortality and serious infection, with significantly higher rates observed in MPA than in patients with GPA (*p* = 0.020 and *p* = 0.024, respectively). In contrast, serotype classification did not exhibit statistically significant differences between MPO-ANCA and PR3-ANCA groups for these outcomes in crude comparisons (*p* = 0.080 and *p* = 0.789, respectively). Although trends towards higher severe relapse rates were observed in the GPA and PR3-ANCA-positive groups, RFS curves were generally comparable between subgroups in both classification systems (Fig. 1).

**Table 1:** Baseline characteristics and outcomes by phenotype and serotype classification in AAV

	Phenotype classification			Serotype classification		
	MPA (*n*=557)	GPA (*n*=172)	P-value (MPA vs. GPA)	MPO (*n*=650)	PR3 (*n*=79)	P-value (MPO vs. PR3)
Age, years	76.00 [69.00, 82.00]	73.00 [64.50, 77.50]	<0.001^*^	76.00 [70.00, 81.00]	65.00 [53.00, 73.00]	<0.001^*^
*Sex*						
Female	325 (58.3)	96 (56.1)	0.672	383 (59.0)	38 (48.1)	0.083
Male	232 (41.7)	75 (43.9)		266 (41.0)	41 (51.9)	
*Phenotype*						
MPA	557 (100.0)	0 (0.0)	<0.001^*^	541 (83.2)	16 (20.3)	<0.001^*^
GPA	0 (0.0)	172 (100.0)		109 (16.8)	63 (79.7)	
Serotype						
MPO	541 (97.1)	109 (63.4)	<0.001^*^	650 (100.0)	0 (0.0)	<0.001^*^
PR3	16 (2.9)	63 (36.6)		0 (0.0)	79 (100.0)	
Follow-up, days	878.00 [259.00, 1590.00]	1257.50 [573.00, 1801.25]	<0.001^*^	943.50 [308.75, 1615.00]	1102.00 [431.50, 1827.00]	0.187
*Organ involvement*						
Constitutional	325 (58.3)	88 (51.2)	0.115	379 (58.3)	34 (43.0)	0.014^*^
Musculoskeletal	227 (40.8)	52 (30.2)	0.017^*^	252 (38.8)	27 (34.2)	0.503
Skin	103 (18.5)	34 (19.8)	0.793	117 (18.0)	20 (25.3)	0.156
Mucosa	6 (1.1)	5 (2.9)	0.173	6 (0.9)	5 (6.3)	0.001^*^
Eyes	30 (5.4)	40 (23.3)	<0.001^*^	49 (7.5)	21 (26.6)	<0.001^*^
Lung	231 (41.5)	100 (58.1)	<0.001^*^	289 (44.5)	42 (53.2)	0.178
Cardiovascular	29 (5.2)	6 (3.5)	0.473	30 (4.6)	5 (6.3)	0.694
Gastrointestinal	7 (1.3)	4 (2.3)	0.517	8 (1.2)	3 (3.8)	0.201
Kidney	442 (79.4)	93 (54.1)	<0.001^*^	488 (75.1)	47 (59.5)	0.005^*^
Central nervous system	47 (8.4)	27 (15.7)	0.009^*^	64 (9.8)	10 (12.7)	0.559
Peripheral nervous system	116 (20.8)	31 (18.0)	0.489	133 (20.5)	14 (17.7)	0.671
BVAS	15.00 [11.00, 19.00]	14.00 [10.00, 20.00]	0.804	15.00 [11.00, 19.00]	15.00 [9.00, 20.50]	0.862
*Laboratory*						
Cre, mg/dL	1.06 [0.70, 1.90]	0.72 [0.59, 0.92]	<0.001^*^	0.96 [0.68, 1.73]	0.71 [0.60, 0.97]	<0.001^*^
CRP, mg/dL	8.25 [2.46, 13.00]	7.98 [2.18, 13.32]	0.822	8.21 [2.62, 13.00]	7.17 [1.26, 14.21]	0.593
KL-6, U/mL	260.00 [172.00, 437.50]	229.00 [159.00, 276.00]	0.005^*^	250.00 [172.00, 418.00]	211.50 [149.00, 269.00]	0.011^*^
Outcome						
Death	74 (13.3)	11 (6.4)	0.020^*^	81 (12.5)	4 (5.1)	0.080
Composite Renal Outcome ^a^	72 (12.9)	14 (8.1)	0.117	78 (12.0)	8 (10.1)	0.762
Severe Relapse^b^	26 (4.7)	13 (7.6)	0.201	32 (4.9)	7 (8.9)	0.229
Serious Infection^c^	105 (19.0)	19 (11.1)	0.024^*^	112 (17.3)	12 (15.4)	0.789

Data are presented as median [interquartile range] for continuous variables or n (%) for categorical variables. *p*-values compare MPA vs. GPA and MPO vs. PR3 using Mann-Whitney U test for continuous variables and chi-square or Fisher’s exact test for categorical variables, as appropriate

*AAV* ANCA-associated vasculitis, *ANCA* anti-neutrophil cytoplasmic antibody, *BVAS* Birmingham Vasculitis Activity Score, *Cre* creatinine, *CRP* C-reactive protein, *GPA* granulomatosis with polyangiitis, *IQR* interquartile range, *KL-6* Krebs von den Lungen-6, *MPA* microscopic polyangiitis, *MPO* myeloperoxidase, *PR3* proteinase 3

*p<0.05

^a^Composite renal outcome was defined as end-stage kidney disease, initiation of renal replacement therapy, or sustained ≥50% decline in estimated glomerular filtration rate from baseline for at least three months

^b^Severe relapse was defined as organ-threatening or life-threatening disease as described in the EULAR recommendations for the management of ANCA-associated vasculitis (2022 update)

^c^Serious infection was defined as infection requiring hospitalization or intravenous antimicrobial therapy

### Comparison of four subgroups based on combined phenotype-serotype classification

We further compared the baseline clinical characteristics and laboratory findings among four combined subgroups (MPO-MPA, PR3-MPA, MPO-GPA, and PR3-GPA). Detailed comparisons are presented in Table 2.

### Comparison within the MPA phenotype: MPO-ANCA vs. PR3-ANCA

Within the MPA phenotype, patients who were MPO-ANCA positive (*n* = 541) were significantly more likely to be female than those positive for PR3-ANCA (*n* = 16) (59.1% vs. 31.2%, *p* = 0.048). Other baseline characteristics, including age, patterns of organ involvement, baseline disease activity, and laboratory parameters (serum creatinine, CRP, and KL-6), were generally comparable between the two MPA subgroups. However, patients with MPO-MPA exhibited non-significant trends towards more frequent constitutional symptoms and lung involvement (Table 2).

### Comparison within the GPA phenotype: MPO-ANCA vs. PR3-ANCA

Within the GPA phenotype, significant baseline differences were observed. MPO-ANCA-positive patients (*n* = 63) were significantly older than PR3-ANCA-positive patients (median age: 75.0 vs. 64.0 years, *p* < 0.001). Furthermore, patients with PR3-GPA had significantly higher frequencies of mucosal (7.9% vs. 0%, *p* = 0.012) and eye involvement (33.3% vs. 17.4%, *p* = 0.028) than those of patients with MPO-GPA. Baseline BVAS, serum creatinine, and CRP levels were similar between the two GPA subgroups. Patients with MPO-GPA showed a non-significant trend towards higher KL-6 levels (*p* = 0.083) (Table 2).

**Table 2:** Comparison of AAV subgroups based on combined phenotype and serotype

	MPO-MPA (*n*=541)	PR3-MPA (*n*=16)	*P*-value (MPO-MPA vs. PR3-MPA)	MPO-GPA (*n*=109)	PR3-GPA (*n*=63)	*P*-value (MPO-GPA vs. PR3-GPA)
Age, years	76.00 [69.00, 82.00]	71.00 [63.75, 80.00]	0.226	75.00 [70.00, 80.00]	64.00 [52.00, 72.00]	<0.001^*^
*Sex*						
Female	320 (59.1)	5 (31.2)	0.048^*^	63 (58.3)	33 (52.4)	0.551
Male	221 (40.9)	11 (68.8)		45 (41.7)	30 (47.6)	
Follow-up, days	882.00 [278.00, 1590.00]	441.50 [51.75, 1640.75]	0.333	1289.00 [580.00, 1709.00]	1175.00 [586.50, 1853.50]	0.976
*Organ involvement*						
Constitutional	319 (59.0)	6 (37.5)	0.145	60 (55.0)	28 (44.4)	0.237
Musculoskeletal	223 (41.2)	4 (25.0)	0.297	29 (26.6)	23 (36.5)	0.234
Skin	99 (18.3)	4 (25.0)	0.724	18 (16.5)	16 (25.4)	0.226
Mucosa	6 (1.1)	0 (0.0)	1.000	0 (0.0)	5 (7.9)	0.012^*^
Eyes	30 (5.5)	0 (0.0)	0.684	19 (17.4)	21 (33.3)	0.028^*^
Lung	228 (42.1)	3 (18.8)	0.106	61 (56.0)	39 (61.9)	0.548
Cardiovascular	27 (5.0)	2 (12.5)	0.446	3 (2.8)	3 (4.8)	0.794
Gastrointestinal	7 (1.3)	0 (0.0)	1.000	1 (0.9)	3 (4.8)	0.277
Kidney	429 (79.3)	13 (81.2)	1.000	59 (54.1)	34 (54.0)	1.000
Central nervous system	46 (8.5)	1 (6.2)	1.000	18 (16.5)	9 (14.3)	0.865
Peripheral nervous system	114 (21.1)	2 (12.5)	0.603	19 (17.4)	12 (19.0)	0.952
BVAS	15.00 [11.00, 20.00]	12.00 [9.75, 15.75]	0.209	13.00 [10.00, 19.00]	16.00 [8.00, 21.50]	0.402
*Laboratory*						
Cre, mg/dL	1.06 [0.70, 1.92]	1.00 [0.76, 1.22]	0.753	0.74 [0.61, 0.94]	0.65 [0.57, 0.85]	0.145
CRP, mg/dL	8.31 [2.65, 13.02]	4.06 [1.13, 9.97]	0.120	7.94 [2.58, 12.40]	8.00 [1.69, 14.54]	0.676
KL-6, U/mL	260.00 [172.00, 442.50]	257.50 [226.25, 299.75]	0.648	236.00 [184.00, 304.00]	193.00 [149.00, 268.25]	0.083
*Outcome*						
Death	73 (13.5)	1 (6.2)	0.640	8 (7.3)	3 (4.8)	0.732
Composite Renal Outcome^a^	72 (13.3)	0 (0.0)	0.236	6 (5.5)	8 (12.7)	0.170
Severe Relapse^b^	26 (4.8)	0 (0.0)	0.767	6 (5.5)	7 (11.1)	0.298
Serious Infection^c^	100 (18.6)	5 (33.3)	0.268	12 (11.1)	7 (11.1)	1.000

Data are presented as median [interquartile range] for continuous variables or n (%) for categorical variables. *p*-values compare MPA vs. GPA and MPO vs. PR3 using Mann-Whitney U test for continuous variables and chi-square or Fisher’s exact test for categorical variables, as appropriate

AAV ANCA-associated vasculitis, *ANCA* anti-neutrophil cytoplasmic antibody, *BVAS* Birmingham Vasculitis Activity Score, *Cre* creatinine, *CRP* C-reactive protein, *GPA* granulomatosis with polyangiitis, *IQR* interquartile range, *KL-6* Krebs von den Lungen-6, *MPA* microscopic polyangiitis, *MPO* myeloperoxidase, *PR3* proteinase 3

*p<0.05

^a^Composite renal outcome was defined as end-stage kidney disease, initiation of renal replacement therapy, or sustained ≥50% decline in estimated glomerular filtration rate from baseline for at least three months

^b^Severe relapse was defined as organ-threatening or life-threatening disease as described in the EULAR recommendations for the management of ANCA-associated vasculitis (2022 update)

^c^Serious infection was defined as infection requiring hospitalization or intravenous antimicrobial therapy

### Survival analysis of combined subgroups

**Fig. 2:**
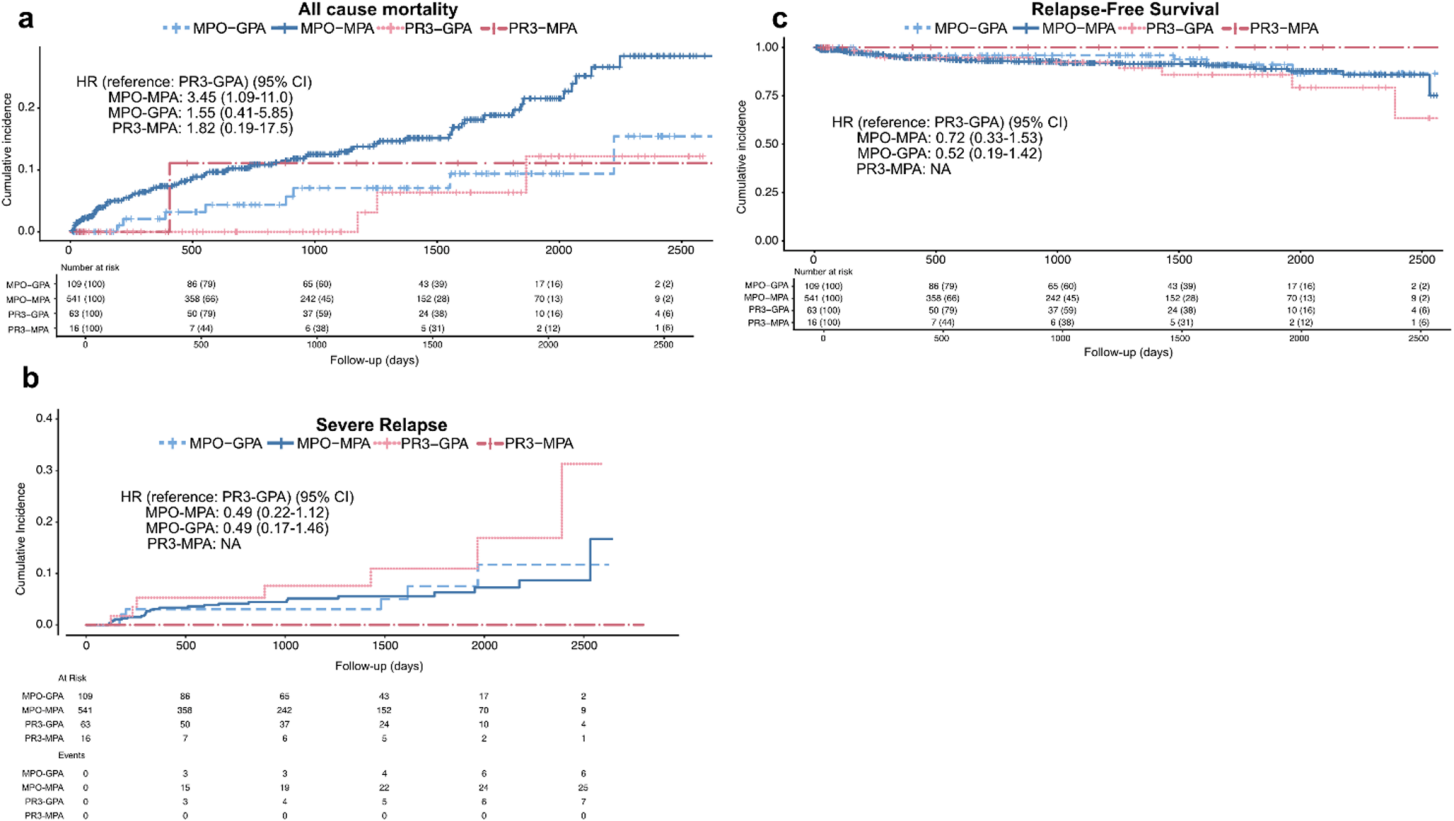
Survival and relapse outcomes according to combined classification in newly diagnosed AAV.** a** All-cause mortality (KM curves), **b** Severe relapse (CIF curves accounting for death as a competing risk),** c** Relapse-free survival (KM curves; event defined as death or severe relapse). All panels are stratified by combined classification groups (MPO-MPA, MPO-GPA, PR3-MPA, and PR3-GPA). Crude HRs with 95% CI, comparing each group to PR3-GPA (reference), and numbers at risk are presented

Survival and relapse outcomes were compared across the four combined subgroups (MPO-MPA, PR3-MPA, MPO-GPA, and PR3-GPA), revealing distinct patterns (Fig. 2). For all-cause mortality, the MPO-MPA subgroup exhibited the highest cumulative incidence, while the other three subgroups showed comparatively lower rates (Fig. 2a). In contrast, the cumulative incidence of severe relapse was highest in the PR3-GPA subgroup. Patients with MPO-MPA and MPO-GPA experienced similarly lower relapse rates, with very few relapses observed in the PR3-MPA subgroup (Fig. 2b; Table 2). Consistent with these findings, RFS was lowest in the PR3-GPA subgroup, whereas patients with MPO-MPA and MPO-GPA demonstrated comparable and higher RFS rates (Fig. 2c).

### Treatment response

**Fig. 3:**
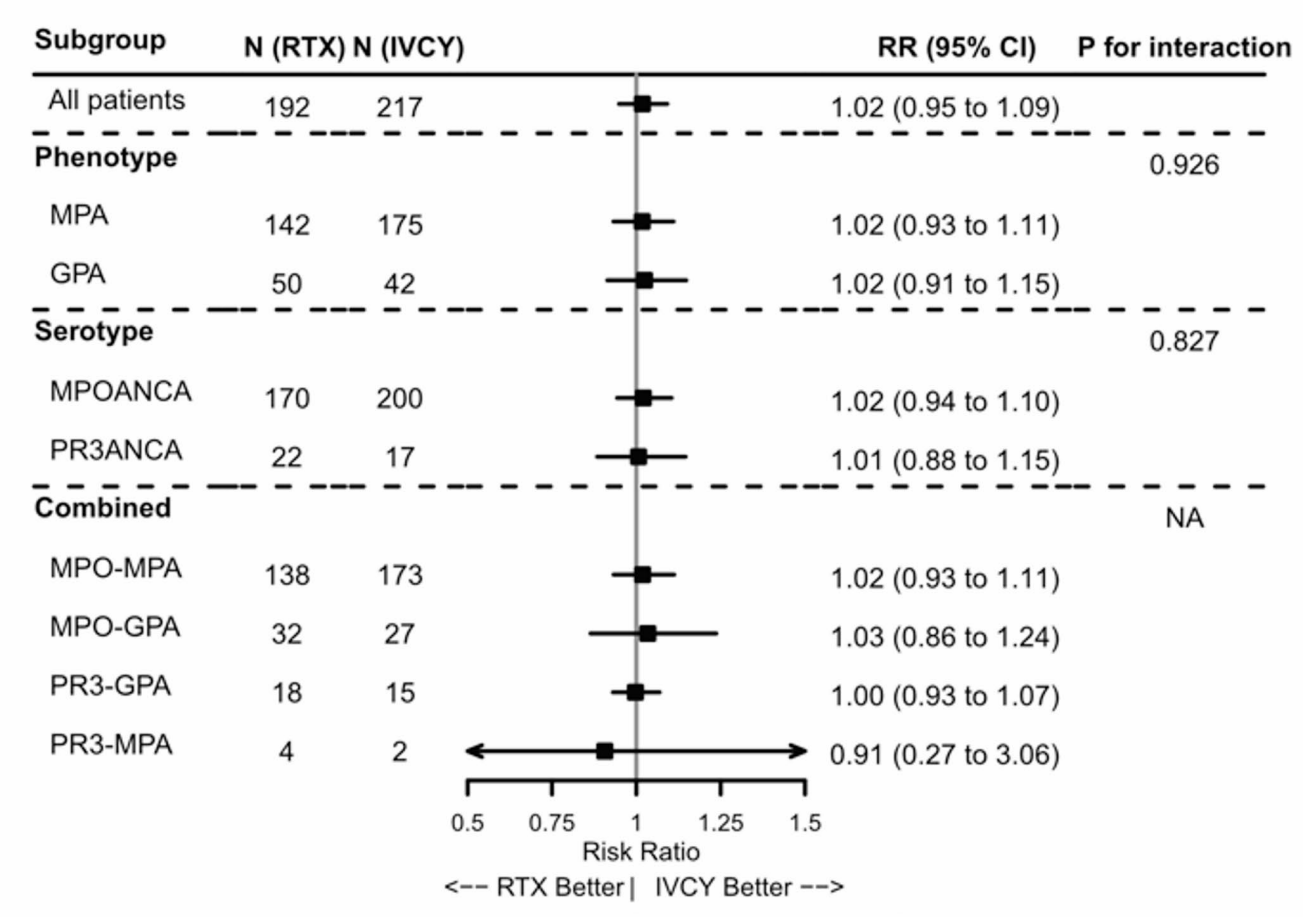
Forest plot of treatment effects on 24-week remission by subgroup classification. Forest plot displaying RRs and 95% CIs comparing IVCY and RTX (reference) for 24-week remission in newly diagnosed patients with AAV receiving either therapy. Estimates are based on IPW-adjusted models applied to multiple imputed datasets. Results are shown overall and stratified by phenotype, serotype, and combined classifications. Squares indicate RRs, and lines represent 95% CIs. The number of patients per treatment arm and p-values for interaction tests (phenotype and serotype only; interaction tests were not performed for other subgroups) are shown.* AAV* ANCA-associated vasculitis, * ANCA* antineutrophil cytoplasmic antibody, * CI* confidence interval, * GPA* granulomatosis with polyangiitis, * IPW* inverse probability of treatment weighting, *IVCY* intravenous cyclophosphamide, *MPA* microscopic polyangiitis, *MPO* myeloperoxidase, *NA* not available, *PR3* proteinase 3, *RR* risk ratio, *RTX* rituximab

The comparative effectiveness of induction therapies (IVCY vs. RTX) on 24-week remission was assessed using IPW-adjusted models (Fig. 3; Online Resource 1, Supplementary Table 3, Supplementary Figs. 3 and 4). Overall, no significant difference in remission rates was observed between the IVCY and RTX groups. Stratified analyses revealed similar non-significant results within phenotype, serotype, or the combined subgroups of MPO-MPA, MPO-GPA, PR3-GPA, or PR3-MPA (Fig. 3). No evidence of treatment effect modification by phenotype or serotype was detected.

### Data-driven clustering analysis

**Fig. 4:**
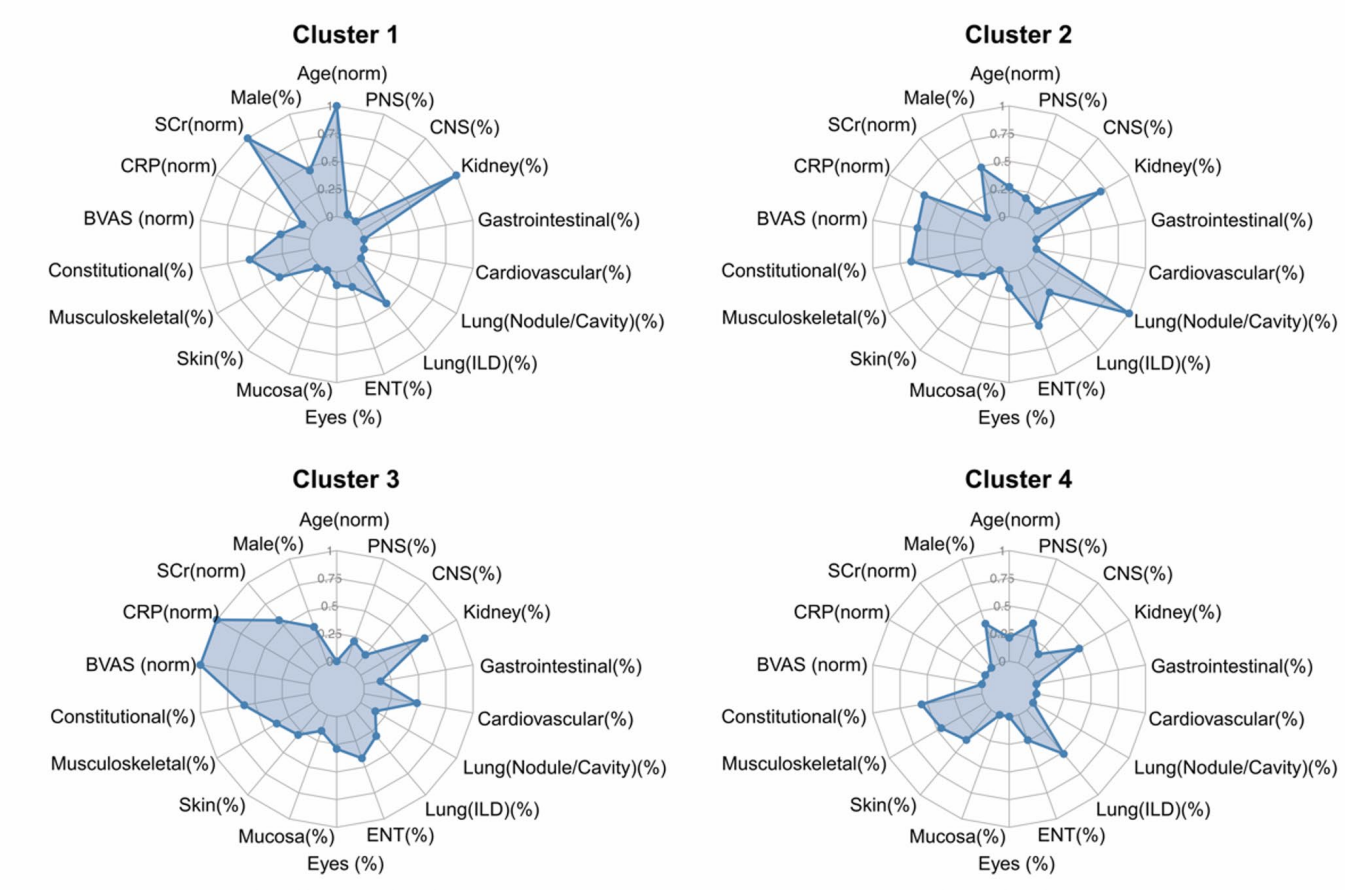
Clinical profiles of data-driven patient clusters. Radar charts showing the profiles of the four clusters identified via data-driven analysis. The axes represent key baseline variables (demographics, employment status, activity, and organ involvement). The value plotted on each axis is guided by the background grid, which uses a proportion scale (0.0 at the centre to 1.0 at the edge). The specific unit for each axis is defined by its label: Axes labelled with (%): These represent categorical variables. The value indicates the percentage of patients within that cluster who have the feature (e.g., a value at the 0.50 grid line on the Kidney (%) axis means 50% of patients have kidney involvement), Axes labelled with (norm): These represent continuous variables. The value indicates the cluster’s mean value, normalised across all four clusters (where 0.0 is the minimum mean and 1.0 is the maximum mean).* BVAS* Birmingham Vasculitis Activity Score, * CNS* central nervous system, * CRP* C-reactive protein, * ENT* ear, nose, and throat, * ILD* interstitial lung disease, * PNS* peripheral nervous system, * SCr* serum creatinine

Unsupervised cluster analysis identified four distinct patient clusters based on baseline clinical and laboratory features (Fig. 4; Online Resource 1, Supplementary Tables 4 and Supplementary Figs. 5 and 6). Cluster 1, the largest group, comprised mostly patients with MPO-MPA and included older individuals with ILD and renal involvement. Cluster 2 consisted primarily of younger patients with PR3- and MPO-GPA, characterised by pulmonary nodules or cavities and ENT involvement. Cluster 3 was characterised by elevated inflammatory markers (CRP and BVAS), multisystem involvement (ENT, renal, and cardiovascular), and included some patients with PR3-GPA. Cluster 4 comprised younger patients with low inflammatory markers but frequent systemic, musculoskeletal, and peripheral nervous system symptoms, encompassing patients from various traditional subgroups. Longitudinal outcomes are presented in Online Resource 1, Supplementary Fig. 7. Significant differences among the clusters were observed for all-cause mortality (*p* = 0.032) and composite renal outcomes (*p* < 0.001) (Supplementary Table 4).

Heatmap analysis comparing these data-driven clusters to conventional phenotype-serotype classifications demonstrated limited concordance (Online Resource 1, Supplementary Fig. 8). This suggests that clustering based on clinical and laboratory profiles captures patterns of heterogeneity distinct from those identified by established AAV classifications.

### Sensitivity analyses

The primary findings were robust across several sensitivity analyses. First, repeating the main comparative analyses using the full cohort, including both newly diagnosed and relapsed patients, yielded results consistent with those from the newly diagnosed cohort alone (Online Resource 1, Supplementary Tables 5 and Supplementary Fig. 9). Second, the estimated treatment effect comparing IVCY and RTX for 24-week remission remained stable when using CCA instead of multiple imputations and when applying TMLE rather than IPW. This confirms the robustness of the findings to different approaches for handling missing data and confounding adjustment (Online Resource 1, Supplementary Table 6). Third, when the ANCA serotype was included as an input variable in the clustering analysis, five clusters were identified. This revealed that PR3-ANCA-positive patients formed a relatively homogeneous group, whereas MPO-ANCA-positive patients displayed greater heterogeneity, highlighting the phenotypic diversity within MPO-ANCA-positive AAV (Online Resource 1, Supplementary Figs. 10 and 11).

## Discussion

This study addresses the ongoing debate regarding the optimal classification strategy for AAV, a challenge previously described as an “unmet need for a unifying view of the disease spectrum” [7]. In this extensive Japanese cohort, we discovered that the traditional classification based on phenotype (MPA vs. GPA) and ANCA serotype (MPO vs. PR3) each offer unique prognostic insights, indicating that they are complementary rather than competing. However, neither approach fully captured the heterogeneity among patients. In contrast, a data-driven clustering analysis identified four distinct subgroups, which demonstrated limited concordance with conventional classifications, underscoring the limitations of existing classification frameworks. These findings contribute to the ongoing debate on optimal AAV classification and emphasise the need for approaches that more accurately reflect the clinical diversity of patients.

While newer AAV classification criteria increasingly emphasise ANCA serotype [24, 25], our results highlight the enduring prognostic relevance of traditional phenotype classification. Notably, the phenotype distinction (MPA vs. GPA) more effectively stratified all-cause mortality risk in unadjusted analyses. This is particularly important because, while patients with MPA were on average older than those with GPA, the age difference between the MPO-ANCA and PR3-ANCA groups was even greater (Table 1). Despite this, phenotype classification still stratified mortality risk more effectively, suggesting it captures prognostic information not solely attributable to age. Serotype classification also demonstrated distinct associations with organ involvement patterns and relapse trends. Our finding that the clinicopathological classification serves as a powerful prognostic discriminator aligns with a previous cohort study [26]. However, our large cohort allows for a more nuanced interpretation, suggesting that the two systems are not competing but are, in fact, complementary. Regarding relapse risk, there was no clear superiority of either the phenotype or the serotype classification over the other; instead, the additional value of ANCA serotype lay in its ability to further stratify risk within a given clinicopathological phenotype. In contrast to the previous study that reported limited utility for such a combined approach [26], our findings suggest that this integrated perspective is essential for a comprehensive patient assessment.

In this study, unsupervised clustering identified four distinct clinical subgroups (Fig. 4), which showed limited concordance with traditional phenotype or serotype classifications (Online Resource 1, Supplementary Fig. 8). The emergence of data-driven subgroups distinct from established classifications aligns with findings from other clustering studies in AAV, which have reported variable cluster numbers, ranging from four to seven, depending on the cohort and analytical methods used [11, 27–28]. Collectively, these findings suggest that AAV heterogeneity extends beyond simple binary classification. The limited concordance in our cohort suggests that clinical profile–based clustering reveals heterogeneous dimensions not captured by conventional classification systems. This highlights the limitations of established criteria for research stratification. These robust, clinically-derived endotypes provide multifaceted clinical utility. They define distinct clinical archetypes that may aid diagnostic recognition, and these patterns in turn suggest different underlying biological endotypes that can generate hypotheses for future research. Finally, these different endotypes were associated with distinct clinical outcomes, with statistically significant differences observed in all-cause mortality and composite renal outcomes (Supplementary Table 4). The pattern of this prognostic association may differ from that of other studies, possibly because our analysis included a broad spectrum of both GPA and MPA, in contrast to studies that have found prognostic stratification within a more focused MPA-only cohort [29]. The identification of these robust clinical patterns within a predominantly MPO-ANCA positive cohort is a key finding in itself, demonstrating that the heterogeneity within MPO-AAV is structured and cannot be captured by serotype alone. The clinical relevance of the four identified clusters requires further validation.

Our findings regarding classification comparisons and disease heterogeneity have broader implications for AAV conceptualisation and management. While binary classifications remain practical, they may be inadequate for capturing the full spectrum of patient diversity, particularly among heterogeneous MPO-ANCA-positive patients and those with discordant phenotype-serotype profiles. This highlights the need for more refined stratification approaches and supports the ongoing shift toward precision medicine in AAV [30]. Enhanced stratification may require the integration of multiple data dimensions, including phenotype, serotype, data-driven clusters, and biomarkers, potentially augmented by multi-omics approaches to uncover deeper biological heterogeneity linked to clinical outcomes [31]. Although the direct clinical implications of this descriptive study are preliminary, identifying these distinct patterns lays the groundwork for future research aimed at disentangling AAV heterogeneity to optimise patient care.

This study has a number of limitations. First, its retrospective design and the use of a single-country cohort may limit the generalisability of the findings. Given the known differences in AAV presentation, such as the predominance of MPO-AAV in Japan compared to the PR3-AAV predominance in many European and North American cohorts [32], our findings, including the cluster profiles, may not be directly applicable to other ethnic groups and require validation in diverse populations. However, the large, multicenter nature of our cohort supports the high generalizability of our findings within the Japanese AAV population. Second, the identified clusters require external validation, and the small sizes of certain subgroups, particularly the PR3-MPA group (*n* = 16), have reduced statistical power for these comparisons. Therefore, the results involving these subgroups should be interpreted with extreme caution, and no definitive conclusions should be drawn from them. Third, the pronounced age differences between groups represent a potential source of confounding in our unadjusted survival analyses. Conversely, our comparative effectiveness analyses for treatment response were designed to mitigate such confounding, using an IPW model that successfully balanced for age (Supplementary Table 3). Fourth, given the retrospective, multicentre design, there is potential for information bias, as the quality and completeness of data abstracted from clinical records may have varied between participating institutions. Despite these limitations, the findings emphasise the importance of future research aimed at validating the clinical relevance of these clusters, investigating the biological underpinnings of MPO-AAV heterogeneity (e.g., through multi-omics approaches), and conducting comparative studies across more diverse populations. Such efforts are essential for integrating multi-dimensional data to improve stratification and enable personalised management for this complex and heterogeneous disease.

In conclusion, this large Japanese cohort demonstrated that traditional phenotype and ANCA serotype classifications provide distinct prognostic insights. However, data-driven clustering revealed additional clinical heterogeneity, particularly within the MPO-ANCA-positive group, that was not captured by conventional classification systems. These findings highlight the need for a more integrative approach to patient stratification in AAV. Incorporating phenotypic, serotypic, and data-driven cluster information is essential to deepen our understanding of disease complexity and advance personalised management for this heterogeneous condition.

## Supplementary Information

Below is the link to the electronic supplementary material.

Supplementary Material 1

## Data Availability

The datasets generated and/or analysed in the current study are available from the corresponding author upon reasonable request.
