# Supplementary material for: Phenotype, serotype, and data-driven clustering reveal complementary dimensions of heterogeneity in ANCA-associated vasculitis: a multicentre Japanese cohort (J-CANVAS)
Source: Rheumatol Int. 2025 Nov 12;45(12):272. doi: 10.1007/s00296-025-06014-y (PMC12611989; doi:10.1007/s00296-025-06014-y)
Supplement: Supplementary file 1 — Supplementary Material 1 [file 296_2025_6014_MOESM1_ESM.pdf]

Online Resource 1

Title: Phenotype, serotype, and data-driven clustering reveal complementary dimensions of heterogeneity in ANCA-associated vasculitis: A multicentre Japanese cohort (J-CANVAS)Journal: Rheumatology International

Genki Kidoguchi[1], Yusuke Yoshida[1], Satoshi Omura[2], Daiki Nakagomi[3], Yoshiyuki Abe[4], Makoto Wada[5], Naoho Takizawa[6], Atsushi Nomura[7], Yuji Kukida[8], Naoya Kondo[9], Hirosuke Takagi[10], Koji Endo[11], Naoto Azuma[12], Tohru Takeuchi[13], Shoichi Fukui[14], Kazuro Kamada[15], Ryo Yanai[16], Yusuke Matsuo[17,18] ,Yasuhiro Shimojima[19], Ryo Nishioka[20], Ryota Okazaki[21], Tomoaki Takata[22], Mayuko Moriyama[23], Ayuko Takatani[24,25], Yoshia Miyawaki[26], Tsuyoshi Shirai[27], Hiroaki Dobashi[28], Takafumi Ito[29], Isao Matsumoto[30], Toshihiko Takada[31], Yutaka Kawahito[2], Toshiko Ito-Ihara[32], Takashi Kida[2], Nobuyuki Yajima[16], Takashi Kawaguchi[33], Shintaro Hirata[1]

[1] Department of Clinical Immunology and Rheumatology, Hiroshima University Hospital, Hiroshima, Japan

[2] Inflammation and Immunology, Graduate School of Medical Science, Kyoto Prefectural University of Medicine, Kyoto, Japan

[3] Department of Rheumatology, University of Yamanashi Hospital, Yamanashi, Japan

[4] Department of Internal Medicine and Rheumatology, Juntendo University, Tokyo, Japan

[5] Center for Rheumatic Disease, Japanese Red Cross Society Kyoto Daiichi Hospital, Kyoto, Japan

[6] Department of Rheumatology, Chubu Rosai Hospital, Aichi, Japan

[7] Immuno-Rheumatology Center, St Luke's International Hospital, Tokyo, Japan

[8] Department of Rheumatology, Japanese Red Cross Society Kyoto Daini Hospital, Kyoto, Japan

[9] Department of Nephrology, Kyoto Katsura Hospital, Kyoto, Japan

[10] Department of Hematology and Rheumatology, Kagoshima University Hospital, Kagoshima, Japan

[11] Department of General internal medicine, Tottori Red Cross Hospital, Tottori, Japan

[12] Department of Diabetes, Endocrinology and Clinical Immunology, Hyogo Medical University School of Medicine, Hyogo, Japan

[13] Department of Internal Medicine (IV), Osaka Medical and Pharmaceutical University, Osaka, Japan

[14] Department of Immunology and Rheumatology, Division of Advanced Preventive Medical Sciences, Nagasaki University Graduate School of Biomedical Sciences, Nagasaki, Japan

[15] Department of Rheumatology, Endocrinology and Nephrology, Faculty of Medicine and Graduate School of Medicine, Hokkaido University, Sapporo, Hokkaido, Japan

[16] Division of Rheumatology, Department of Medicine, Showa Medical University School of Medicine, Tokyo, Japan

[17] Department of Rheumatology, Tokyo Kyosai Hospital, Tokyo, Japan

[18] Department of Rheumatology, Graduate School of Medical and Dental Sciences, Institute of Science Tokyo (formerly Tokyo Medical and Dental University)

[19] Department of Medicine (Neurology and Rheumatology), Shinshu University School of Medicine, Nagano, Japan

[20] Department of Rheumatology, Graduate School of Medical Science, Kanazawa University, Kanazawa, Japan

[21] Division of Respiratory Medicine and Rheumatology, Department of Multidisciplinary Internal Medicine, Faculty of Medicine, Tottori University, Tottori, Japan

[22] Division of Gastroenterology and Nephrology, Tottori University, Tottori, Japan

[23] Department of Rheumatology, Shimane University Faculty of Medicine, Shimane, Japan

[24] Rheumatic Disease Center, Sasebo Chuo Hospital, Nagasaki, Japan

[25] Department of Public Health, Nagasaki University Graduate School of Biomedical Sciences, Nagasaki, Japan

[26] Department of Nephrology, Rheumatology, Endocrinology and Metabolism, Okayama University Graduate School of Medicine, Dentistry and Pharmaceutical Sciences, Okayama, Japan

[27] Department of Rheumatology, Tohoku University Hospital, Sendai, Miyagi, Japan

[28] Division of Hematology, Rheumatology and Respiratory Medicine, Department of Internal Medicine, Faculty of Medicine, Kagawa University, Kagawa, Japan

[29] Division of Nephrology, Department of Internal Medicine, Teikyo University Chiba Medical Center, Chiba, Japan

[30] Department of Rheumatology, Institute of Medicine, University of Tsukuba, Tsukuba, Ibaraki, Japan

[31] Department of General Medicine, Shirakawa Satellite for Teaching And Research (STAR) Fukushima Medical University, Shirakawa, Fukushima, Japan

[32] The Clinical and Translational Research Center, University Hospital, Kyoto Prefectural University of Medicine, Kyoto, Japan

[33] Department of Clinical Assessment, Tokyo University of Pharmacy and Life Sciences, Tokyo, Japan

*Corresponding Author:

Genki Kidoguchi, MD[1]

[1]Department of Clinical Immunology and Rheumatology, Hiroshima University Hospital, 1-2-3 Kasumi, Minami-ku, Hiroshima, 734-8551, Japan

Email: kidogen@hiroshima-u.ac.jp

ORCiD: 0009-0001-0558-8814

Supplementary Data. Supplementary Methods Details

Supplementary Table 1. List of Participating Centers in J-CANVAS

Supplementary Table 2. Summary of Missing Data in the Treatment Comparison Analysis

Supplementary Table 3. Baseline Characteristics Before and After IPW

Supplementary Table 4. Baseline Characteristics and Clinical Outcomes of Identified Clusters

Supplementary Table 5. Results of Sensitivity Analysis Including Relapsed Patients

Supplementary Table 6. Sensitivity Analyses for Treatment Effect Estimation

Supplementary Fig. 1 Directed Acyclic Graph (DAG) for Covariate Selection

Supplementary Fig. 2 Patient Selection Flowchart

Supplementary Fig. 3 Propensity Score Distribution Before and After Weighting

Supplementary Fig. 4 Love Plot for Propensity Score Balance Assessment

Supplementary Fig. 5 BIC Plot for Determining the Optimal Number of Clusters

Supplementary Fig. 6 Visualization of Identified Clusters using mclustDR

Supplementary Fig. 7 Survival and Relapse Outcomes for the Four Data-Driven Clusters

Supplementary Fig. 8 Concordance between Data-Driven Clusters and Conventional Classifications in the Primary Analysis

Supplementary Fig. 9 Relapse-Free Survival: Sensitivity Analysis Including Relapsed Patients

Supplementary Fig. 10 Radar Chart for Clustering: Sensitivity Analysis Including Serotype

Supplementary Fig. 11 Sensitivity Analysis: Concordance between Data-Driven Clusters and Conventional Classifications Including Serotype

Supplementary Data

Covariates in the Propensity Score Model

The propensity score (PS) for receiving rituximab versus intravenous cyclophosphamide was estimated using logistic regression. Covariate selection was guided by clinical importance and a directed acyclic graph. (DAG, Supplementary Fig. S2) The final PS model incorporated the following baseline continuous covariates: age at diagnosis, serum creatinine level, and initial glucocorticoid dose. The model also included these baseline categorical covariates: presence of diabetes mellitus, hypertension, chronic kidney disease (CKD), cardiovascular disease (CVD), cerebrovascular disease, disease phenotype (MPA vs. GPA), ANCA serotype (MPO-ANCA vs. PR3-ANCA), and the presence of organ- or life-threatening manifestations.

Variables Included in the Data-Driven Clustering Analysis

Data-driven clustering analysis, utilizing latent class analysis with a Gaussian finite mixture model, was performed to identify distinct patient subgroups based on baseline characteristics. The final clustering model incorporated several baseline variables. Continuous variables included were age at diagnosis, serum creatinine level, C-reactive protein (CRP) level, and the Birmingham Vasculitis Activity Score (BVAS) version 3.0 total score. The model also included baseline categorical variables, primarily representing presence or absence: sex (female vs. male), constitutional symptoms, musculoskeletal involvement, skin involvement, mucosal involvement (non-eye/ENT), eye involvement, Ear, Nose, Throat (ENT) involvement, cardiovascular involvement, gastrointestinal involvement, renal involvement, central nervous system involvement, and peripheral nervous system involvement. Notably, pulmonary involvement was specifically

classified into two separate categorical variables indicating interstitial lung disease (ILD) or pulmonary nodules/cavities. In line with the main text Methods, disease phenotype (MPA/GPA) and ANCA serotype (MPO/PR3-ANCA) were excluded from this primary analysis to avoid circularity when comparing the resulting clusters with conventional classifications. All variables included in the model were standardized prior to the clustering procedure within each imputed dataset.

Supplementary Table 1. List of Participating Centers in J-CANVAS

No.	Institution and Location
1	Department of Clinical Immunology and Rheumatology, Hiroshima University Hospital, Hiroshima, Japan
2	Inflammation and Immunology, Graduate School of Medical Science, Kyoto Prefectural University of Medicine, Kyoto, Japan
3	Department of Rheumatology, University of Yamanashi Hospital, Yamanashi, Japan
4	Department of Internal Medicine and Rheumatology, Juntendo University, Tokyo, Japan
5	Center for Rheumatic Disease, Japanese Red Cross Society Kyoto Daiichi Hospital, Kyoto, Japan
6	Department of Rheumatology, Chubu Rosai Hospital, Aichi, Japan
7	Immuno-Rheumatology Center, St Luke's International Hospital, Tokyo, Japan
8	Department of Rheumatology, Japanese Red Cross Society Kyoto Daini Hospital, Kyoto, Japan
9	Department of Nephrology, Kyoto Katsura Hospital, Kyoto, Japan
10	Department of Hematology and Rheumatology, Kagoshima University Hospital, Kagoshima, Japan
11	Department of General internal medicine, Tottori Red Cross Hospital, Tottori, Japan
12	Department of Diabetes, Endocrinology and Clinical Immunology, Hyogo Medical University School of Medicine, Hyogo, Japan
13	Department of Internal Medicine (IV), Osaka Medical and Pharmaceutical University, Osaka, Japan
14	Department of Immunology and Rheumatology, Division of Advanced Preventive Medical Sciences, Nagasaki University Graduate School of Biomedical Sciences, Nagasaki, Japan
15	Department of Rheumatology, Endocrinology and Nephrology, Faculty of Medicine and Graduate School of Medicine, Hokkaido University, Sapporo, Hokkaido, Japan
16	Division of Rheumatology, Department of Medicine, Showa University School of Medicine, Tokyo, Japan
17	Department of Rheumatology, Tokyo Kyosai Hospital, Tokyo, Japan
18	Department of Medicine (Neurology and Rheumatology), Shinshu University School of Medicine, Nagano, Japan
19	Department of Rheumatology, Graduate School of Medical Science, Kanazawa University, Kanazawa, Japan
20	Division of Respiratory Medicine and Rheumatology, Department of Multidisciplinary Internal Medicine, Faculty of Medicine, Tottori University, Tottori, Japan
21	Division of Gastroenterology and Nephrology, Tottori University, Tottori, Japan
22	Department of Rheumatology, Shimane University Faculty of Medicine, Shimane, Japan
23	Rheumatic Disease Center, Sasebo Chuo Hospital, Nagasaki, Japan
24	Department of Public Health, Nagasaki University Graduate School of Biomedical Sciences, Nagasaki, Japan
25	Department of Nephrology, Rheumatology, Endocrinology and Metabolism, Okayama University Graduate School of Medicine, Dentistry and Pharmaceutical Sciences, Okayama, Japan
26	Department of Rheumatology, Tohoku University Hospital, Sendai, Miyagi, Japan

27 Division of Hematology, Rheumatology and Respiratory Medicine, Department of Internal Medicine, Faculty of Medicine, Kagawa University, Kagawa, Japan

28 Division of Nephrology, Department of Internal Medicine, Teikyo University Chiba Medical Center, Chiba, Japan

29 Department of Rheumatology, Institute of Medicine, University of Tsukuba, Tsukuba, Ibaraki, Japan

30 Department of General Medicine, Shirakawa Satellite for Teaching And Research (STAR) Fukushima Medical University, Shirakawa, Fukushima, Japan

Supplementary Table 2. Summary of Missing Data in the Treatment Comparison Analysis

Variable	RTX (n=192)	IVCY (n=217)
Age at diagnosis	0(0.0)	0(0.0)
Sex	0(0.0)	0(0.0)
Phenotype	0(0.0)	0(0.0)
Serotype	0(0.0)	0(0.0)
Follow-up days	0(0.0)	0(0.0)
Diabetes	0(0.0)	0(0.0)
Hypertension	0(0.0)	0(0.0)
CKD	0(0.0)	0(0.0)
CVD	0(0.0)	0(0.0)
Cerebrovascular_disease	0(0.0)	0(0.0)
Organ- or life-threatening manifestations	0(0.0)	0(0.0)
Cre	1(0.5)	0(0.0)
CRP	0(0.0)	0(0.0)
Initial GC dose	0(0.0)	0(0.0)
Remission Status at 24wk	29(15.1)	25(11.5)

Data are n (% missing within group).

Abbreviations: CKD, Chronic Kidney Disease; Cre, Serum Creatinine; CRP, C-Reactive Protein; CVD, Cardiovascular Disease; GC, Glucocorticoid; IVCY, Intravenous Cyclophosphamide; RTX, Rituximab; Wk, Week.

Supplementary Table 3. Baseline Characteristics Before and After IPW

		Before weighting			After weighting		
		RTX (n=192)	IVCY (n=217)	SMD	RTX (n=410.10)	IVCY (n=408.17)	SMD
Age, years		75.00 [68.00, 81.00]	74.00 [68.00, 79.00]	0.060	75.16 [69.00, 81.00]	74.00 [68.00, 79.00]	<0.001
Phenotype	MPA	142 (74.0)	175 (80.6)	0.160	318.5 (77.7)	317.5 (77.8)	0.003
	GPA	50 (26.0)	42 (19.4)		91.6 (22.3)	90.6 (22.2)	
Serotype	MPO	170 (88.5)	200 (92.2)	0.123	371.2 (90.5)	369.9 (90.6)	0.004
	PR3	22 (11.5)	17 (7.8)		38.9 (9.5)	38.2 (9.4)	
Diabetes		36 (18.8)	50 (23.0)	0.106	89.3 (21.8)	87.7 (21.5)	0.007
Hypertension		90 (46.9)	103 (47.5)	0.012	194.3 (47.4)	192.8 (47.2)	0.003
CKD		32 (16.7)	32 (14.7)	0.053	65.4 (16.0)	65.6 (16.1)	0.003
CVD		30 (15.6)	31 (14.3)	0.038	60.8 (14.8)	60.3 (14.8)	0.002
Cerebrovascular disease		9 (4.7)	17 (7.8)	0.130	25.4 (6.2)	25.7 (6.3)	0.005
Cre, mg/dL		0.94 [0.72, 1.61]	1.08 [0.70, 1.98]	0.120	0.96 [0.72, 1.84]	1.06 [0.69, 1.80]	0.006
Organ or life-threatening manifestations		110 (57.3)	137 (63.1)	0.120	249.3 (60.8)	247.1 (60.5)	0.005
Initial GC dose, mg		45.00 [30.00, 55.00]	45.00 [40.00, 55.00]	0.060	45.00 [35.00, 55.00]	45.00 [40.00, 55.00]	<0.001

Data are presented as median [interquartile range] for continuous variables or n (%) for categorical variables.

Abbreviations: CKD, Chronic Kidney Disease; Cre, Serum Creatinine; CVD, Cardiovascular Disease; GC, Glucocorticoid; IPW, Inverse Probability of Treatment Weighting; IVCY, Intravenous Cyclophosphamide; RTX, Rituximab; SMD, Standardized Mean Difference; Wk, Week.

Supplementary Table 4. Baseline Characteristics and Clinical Outcomes of Identified Clusters

		Cluster				
		1<br>(n=291)	2<br>(n=88)	3<br>(n=76)	4<br>(n=274)	P-value
Age, years		76.00 [69.00, 82.00]	74.00 [69.75, 80.00]	74.50 [67.00, 81.25]	75.00 [68.00, 80.00]	0.255
Sex	Female	156 (53.6)	45 (51.1)	50 (65.8)	170 (62.3)	0.048*
	Male	135 (46.4)	43 (48.9)	26 (34.2)	103 (37.7)	
Phenotype	GPA	33 (11.3)	60 (68.2)	28 (36.8)	51 (18.6)	<0.001*
	MPA	258 (88.7)	28 (31.8)	48 (63.2)	223 (81.4)	
Serotype	MPO	272 (93.5)	65 (73.9)	59 (77.6)	254 (92.7)	<0.001*
	PR3	19 (6.5)	23 (26.1)	17 (22.4)	20 (7.3)	
Combined	MPO-GPA	22 (7.6)	37 (42.0)	13 (17.1)	37 (13.5)	<0.001*
	MPO-MPA	250 (85.9)	28 (31.8)	46 (60.5)	217 (79.2)	
	PR3-GPA	11 (3.8)	23 26.1)	15 19.7)	14 (5.1)	
	PR3-MPA	8 (2.7)	0 (0.0)	2 (2.6)	6 (2.2)	
Follow-up, days		922.00<br>[290.00, 1590.00]	1078.00<br>[340.75, 1615.00]	680.50<br>[122.00, 1528.50]	1039.50<br>[468.75, 1707.25]	0.041*
Organ involvement						
	Constitutional	160 (55.0)	57 (64.8)	45 (59.2)	151 (55.1)	0.367
	Musculoskeletal	99 (34.0)	25 (28.4)	30 (39.5)	125 (45.6)	0.007*
	Skin	6 (2.1)	11 (12.5)	24 (31.6)	96 (35.0)	<0.001*
	Mucosa	0 (0.0)	0 (0.0)	11 14.5)	0 (0.0)	<0.001*

Eyes	32 (11.0)	13 (14.8)	25 (32.9)	0 (0.0)	<0.001*
Lung	115 (39.5)	88 (100.0)	35 (46.1)	93 (33.9)	<0.001*
Cardiovascular	0 (0.0)	0 (0.0)	35 (46.1)	0 (0.0)	<0.001*
Gastrointestinal	0 (0.0)	0 (0.0)	11 (14.5)	0 (0.0)	<0.001*
Kidney	290 (99.7)	62 (70.5)	52 (68.4)	131 (47.8)	<0.001*
Central nervous system	2 (0.7)	13 (14.8)	15 (19.7)	44 (16.1)	<0.001*
Peripheral nervous system	9 (3.1)	17 (19.3)	17 (22.4)	104 (38.0)	<0.001*
BVAS	14.00 [12.00, 17.00]	17.50 [13.00, 21.00]	21.00 [15.75, 26.00]	13.00 [6.00, 19.00]	<0.001*
Laboratory					
Cre, mg/dL	1.30 [0.81, 2.42]	0.78 [0.63, 1.19]	0.90 [0.62, 1.65]	0.75 [0.60, 1.11]	<0.001*
CRP, mg/dL	7.90 [2.03, 13.20]	9.47 [3.91, 12.40]	9.43 [4.28, 13.77]	7.83 [2.54, 12.60]	0.228
KL-6, U/mL	254.00 [175.00, 429.50]	236.50 [196.00, 311.25]	209.00 [149.00, 278.00]	252.00 [166.00, 438.00]	0.141
Outcome					
Death	46 (15.8)	6 (6.8)	6 (7.9)	27 (9.9)	0.032*
Composite Renal Outcome[a]	49 (16.8)	9 (10.2)	12 (15.8)	16 (5.8)	<0.001*
Severe Relapse[b]	11 (3.8)	9 (10.2)	4 (5.3)	15 (5.5)	0.135
Serious Infection[c]	62 (21.4)	10 (11.4)	13 (17.3)	39 (14.3)	0.064

Data are presented as median [interquartile range] for continuous variables or n (%) for categorical variables. *p*-values compare MPA vs GPA and MPO vs PR3 using Mann-Whitney U test for continuous variables and chi-square or Fisher's exact test for categorical variables, as appropriate. Significance level was set at $p<0.05$.

Abbreviations: AAV, ANCA-associated vasculitis; ANCA, anti-neutrophil cytoplasmic antibody; BVAS, Birmingham Vasculitis Activity Score; Cre, creatinine; CRP, C-reactive protein; GPA, granulomatosis with polyangiitis; IQR, interquartile range; KL-6, Krebs von den Lungen-6; MPA, microscopic polyangiitis; MPO, myeloperoxidase; PR3, proteinase 3

*p<0.05.

[a]Composite renal outcome was defined as end-stage kidney disease, initiation of renal replacement therapy, or sustained ≥50% decline in estimated glomerular filtration rate from baseline for at least three months.

[b]Severe relapse was defined as organ-threatening or life-threatening disease as described in the EULAR recommendations for the management of ANCA-associated vasculitis (2022 update).

[c]Serious infection was defined as infection requiring hospitalization or intravenous antimicrobial therapy.

Supplementary Table 5. Results of Sensitivity Analysis Including Relapsed Patients

		Phenotype Classification			Serotype Classification		
		MPA (n=640)	GPA (n=234)	P-value (MPA vs GPA)	MPO (n=750)	PR3 (n=124)	P-value (MPO vs PR3)
Age, years		76.00 [69.00, 81.00]	72.00 [61.00, 77.00]	<0.001*	76.00 [70.00, 81.00]	64.00 [51.00, 73.00]	<0.001*
Sex	Female	365 (57.0)	130 (55.8)	0.803	434 (57.9)	61 (49.2)	0.085
	Male	275 (43.0)	103 (44.2)		315 (42.1)	63 (50.8)	
Status	New	557 (87.0)	172 (73.5)	<0.001*	650 (86.7)	79 (63.7)	<0.001*
	Relapse	83 (13.0)	62 (26.5)		100 (13.3)	45 (36.3)	
Phenotype	MPA	640 (100.0)	0 (0.0)	<0.001*	621 (82.8)	19 (15.3)	<0.001*
	GPA	0 (0.0)	234 (100.0)		129 (17.2)	105 (84.7)	
Serotype	MPO	621 (97.0)	129 (55.1)	<0.001*	750 (100.0)	0 (0.0)	<0.001*
	PR3	19 (3.0)	105 (44.9)		0 (0.0)	124 (100.0)	
Follow-up, days		840.00 [253.25, 1574.25]	1288.00 [633.50, 1885.75]	<0.001*	926.00 [300.25, 1612.75]	1232.50 [584.00, 1919.00]	0.001*
Organ involvement							
	Constitutional	340 (53.1)	95 (40.6)	0.001*	397 (52.9)	38 (30.6)	<0.001*
	Musculoskeletal	239 (37.3)	57 (24.4)	<0.001*	267 (35.6)	29 (23.4)	0.010*
	Skin	107 (16.7)	36 (15.4)	0.712	122 (16.3)	21 (16.9)	0.956
	Mucosa	6 (0.9)	7 (3.0)	0.057	7 (0.9)	6 (4.8)	0.003*
	Eyes	36 (5.6)	49 (20.9)	<0.001*	58 (7.7)	27 (21.8)	<0.001*
	Lung	258 (40.3)	128 (54.7)	<0.001*	323 (43.1)	63 (50.8)	0.131
	Cardiovascular	31 (4.8)	7 (3.0)	0.317	33 (4.4)	5 (4.0)	1.000

	Gastrointestinal	7 (1.1)	5 (2.1)	0.398	8 (1.1)	4 (3.2)	0.134
	Kidney	490 (76.6)	112 (47.9)	<0.001*	545 (72.7)	57 (46.0)	<0.001*
	Central nervous system	63 (9.8)	42 (17.9)	0.002*	83 (11.1)	22 (17.7)	0.049*
	Peripheral nervous system	132 (20.6)	41 (17.5)	0.356	152 (20.3)	21 (16.9)	0.459
	BVAS	14.00 [10.00, 19.00]	12.00 [8.00, 19.00]	0.054	14.00 [10.00, 19.00]	12.00 [7.00, 18.00]	0.018*
Laboratory							
	Cre, mg/dL	1.06 [0.71, 1.90]	0.72 [0.59, 0.92]	<0.001*	0.96 [0.69, 1.70]	0.71 [0.60, 1.00]	<0.001*
	CRP, mg/dL	7.40 [1.96, 12.44]	6.44 [1.34, 12.10]	0.112	7.47 [2.14, 12.45]	3.66 [0.57, 10.65]	0.002*
	KL-6, U/mL	275.00 [179.50, 465.00]	227.50 [159.00, 272.00]	<0.001*	261.00 [180.75, 446.00]	199.00 [151.00, 269.00]	<0.001*
Outcome							
	Death	88 (13.8)	16 (6.8)	0.007*	96 (12.8)	8 (6.5)	0.061
	Composite Renal Outcome [a]	83 (13.0)	16 (6.8)	0.016*	89 (11.9)	10 (8.1)	0.278
	Severe Relapse[b]	33 (5.2)	19 (8.1)	0.139	39 (5.2)	13 (10.5)	0.036*
	Serious Infection[c]	125 (19.7)	33 (14.2)	0.083	136 (18.2)	22 (18.0)	1.000

Data are presented as median [interquartile range] for continuous variables or n (%) for categorical variables. *p*-values compare MPA vs GPA and MPO vs PR3 using Mann-Whitney U test for continuous variables and chi-square or Fisher's exact test for categorical variables, as appropriate. Significance level was set at p<0.05.

Abbreviations: AAV, ANCA-associated vasculitis; ANCA, anti-neutrophil cytoplasmic antibody; BVAS, Birmingham Vasculitis Activity Score; Cre, creatinine; CRP, C-reactive protein; GPA, granulomatosis with polyangiitis; IQR, interquartile range; KL-6, Krebs von den Lungen-6; MPA, microscopic polyangiitis; MPO, myeloperoxidase; PR3, proteinase 3.

*p<0.05.

[a]Composite renal outcome was defined as end-stage kidney disease, initiation of renal replacement therapy, or sustained ≥50% decline in estimated glomerular filtration rate from baseline for at least three months.

[b]Severe relapse was defined as organ-threatening or life-threatening disease as described in the EULAR recommendations for the management of ANCA-associated vasculitis (2022 update).

[c]Serious infection was defined as infection requiring hospitalization or intravenous antimicrobial therapy.

Supplementary Table 6. Sensitivity Analyses for Treatment Effect Estimation

Analysis approach	Dataset	Risk Ratio	95% CI
Primary Analysis			
Multiple Imputation + IPW	Imputed (N=409)	1.02	0.947 to 1.096
Sensitivity Analysis			
Complete Case + IPW	Complete Case (N=351)	1.01	0.943 to 1.084
Complete Case + DRE(TMLE)	Complete Case (N=351)	1.01	0.945 to 1.082

Abbreviations: CI, confidence interval; DRE, double robust estimation; IPW, inverse probability of treatment weighting; TMLE, targeted maximum likelihood estimation.

This table presents the estimated Risk Ratio (RR) and 95% Confidence Interval (CI) for achieving remission at 24 weeks comparing rituximab versus intravenous cyclophosphamide. Results from the primary analysis, which utilized multiple imputation (MI) and inverse probability of treatment weighting (IPW), are compared with results from two sensitivity analyses conducted on the complete case dataset: one using IPW and another using double robust estimation (specifically, targeted maximum likelihood estimation [DRE (TMLE)]).

Supplementary Fig. 1 Directed Acyclic Graph (DAG) for Covariate Selection

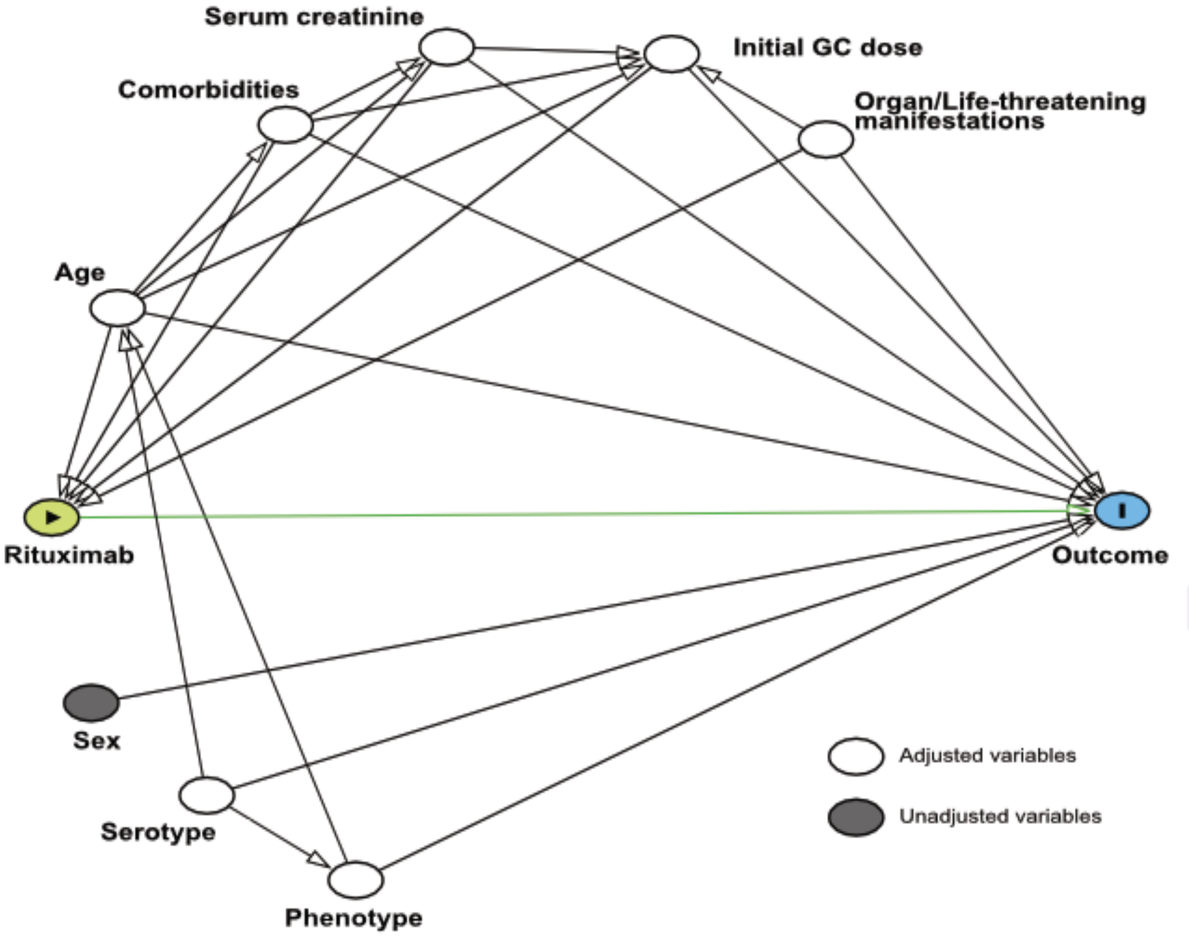

This DAG depicts the hypothesized causal relationships between baseline patient characteristics, treatment assignment (Rituximab [exposure, highlighted yellow, indicated by symbol] versus Intravenous Cyclophosphamide [comparator, not explicitly shown]), and the primary outcome (remission at 24 weeks [highlighted blue]). Arrows indicate assumed causal effects between variables. This causal structure was used to identify potential confounding factors and guide the selection of covariates included in the propensity score model for adjusting the estimated treatment effect.

- **Comorbidities:** This node represents the presence of one or more of the following pre-existing conditions recorded at baseline: diabetes mellitus, hypertension, chronic kidney disease (CKD), cardiovascular disease (CVD), and cerebrovascular disease.
- **Organ or life-threatening manifestations:** This node indicates the presence at baseline of severe disease activity, defined based on the EULAR 2022 recommendations for management of ANCA-associated vasculitis. Specific features included, among others: biopsy-proven glomerulonephritis, pulmonary hemorrhage, cardiomyopathy, major neurological involvement (such as meningitis, altered consciousness, seizure, stroke, cranial neuropathy, sensory or motor peripheral neuropathy), specific ophthalmologic involvement (like proptosis or vision-threatening disease), and major abdominal events (like peritonitis or ischemic bowel disease).

Supplementary Fig. 2 Patient Selection Flowchart

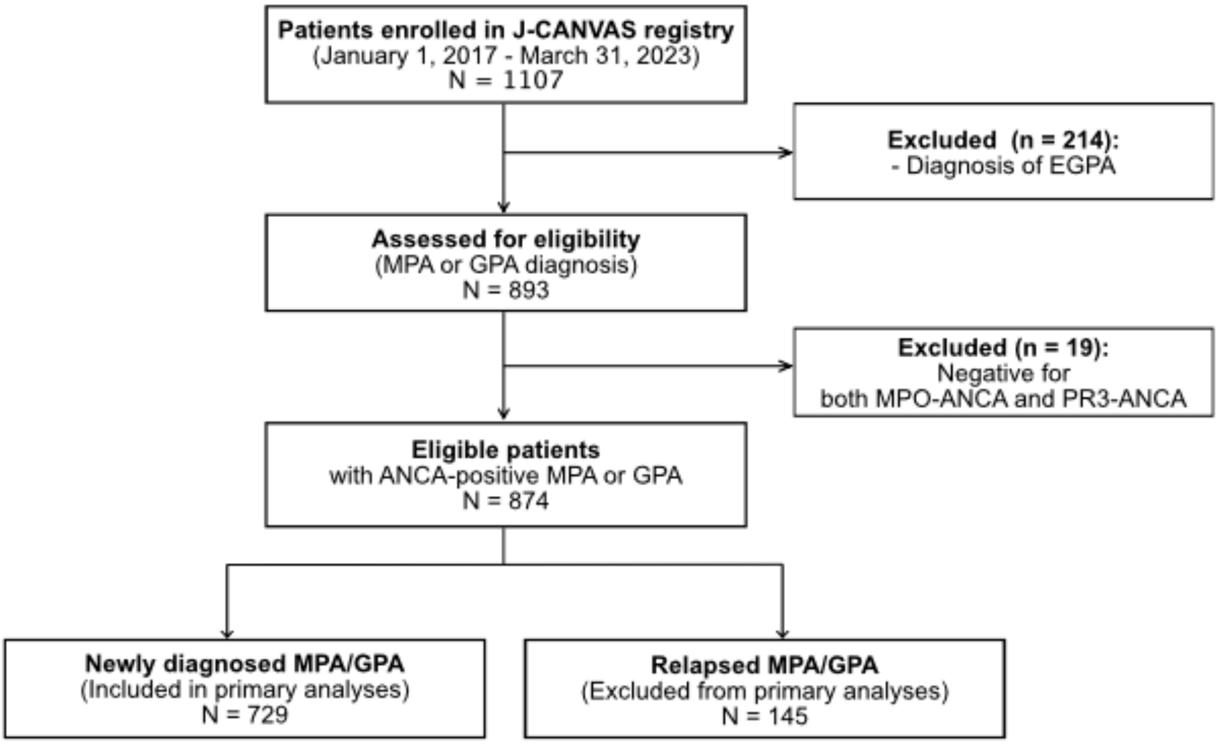

This diagram shows the process of selecting eligible patients with ANCA-positive MPA or GPA from the J-CANVAS registry (enrollment period: January 1, 2017 - March 31, 2023), detailing the exclusion criteria applied and the final number of patients included in the analyses, stratified by newly diagnosed and relapsed cases.

Abbreviations: ANCA, Anti-Neutrophil Cytoplasmic Antibody; EGPA, Eosinophilic granulomatosis with polyangiitis; GPA, Granulomatosis with polyangiitis; J-CANVAS, Japan Collaborative Registry of ANCA-Associated Vasculitis; MPA, Microscopic polyangiitis; MPO-ANCA, Myeloperoxidase Anti-Neutrophil Cytoplasmic Antibody; PR3-ANCA, Proteinase 3 Anti-Neutrophil Cytoplasmic Antibody.

Supplementary Fig. 3 Propensity Score Distribution Before and After Weighting

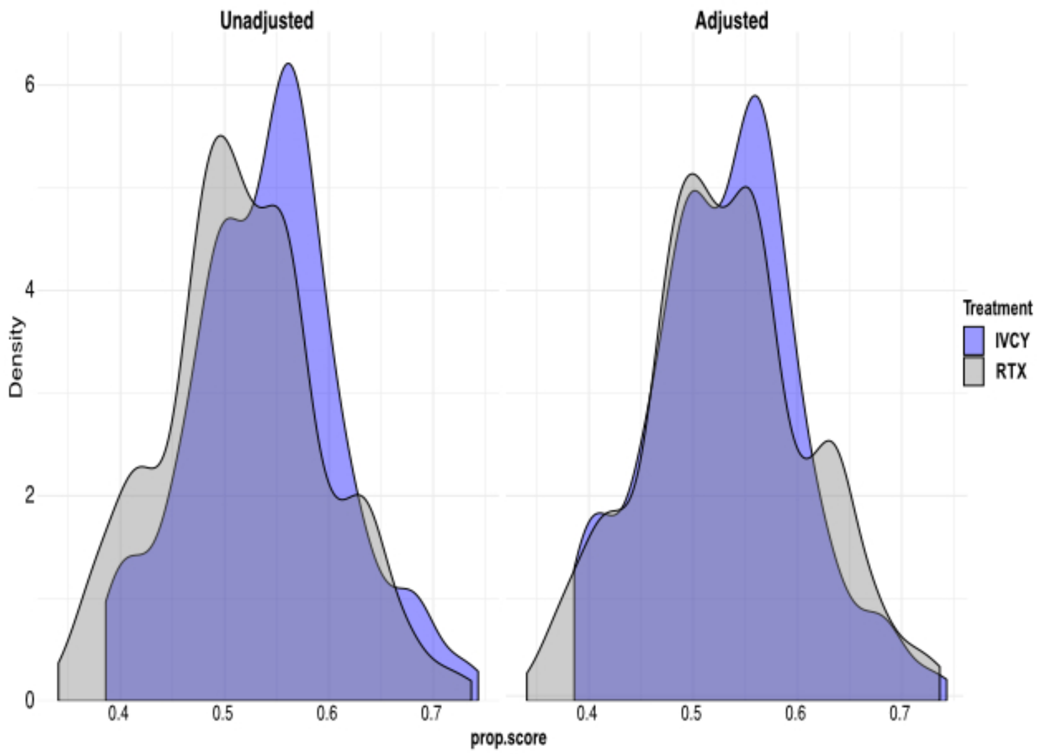

Density distribution of propensity scores by treatment group (RTX vs IVCY). The left panel shows the distribution before inverse probability of treatment weighting (IPW) (Unadjusted Sample), and the right panel shows the distribution after weighting (Adjusted Sample). The greater similarity in distributions after weighting indicates improved propensity score balance between groups achieved by IPW. Results are based on the first imputed dataset.

Abbreviations: IVCY, Intravenous Cyclophosphamide; RTX, Rituximab

Supplementary Fig. 4 Love Plot for Propensity Score Balance Assessment

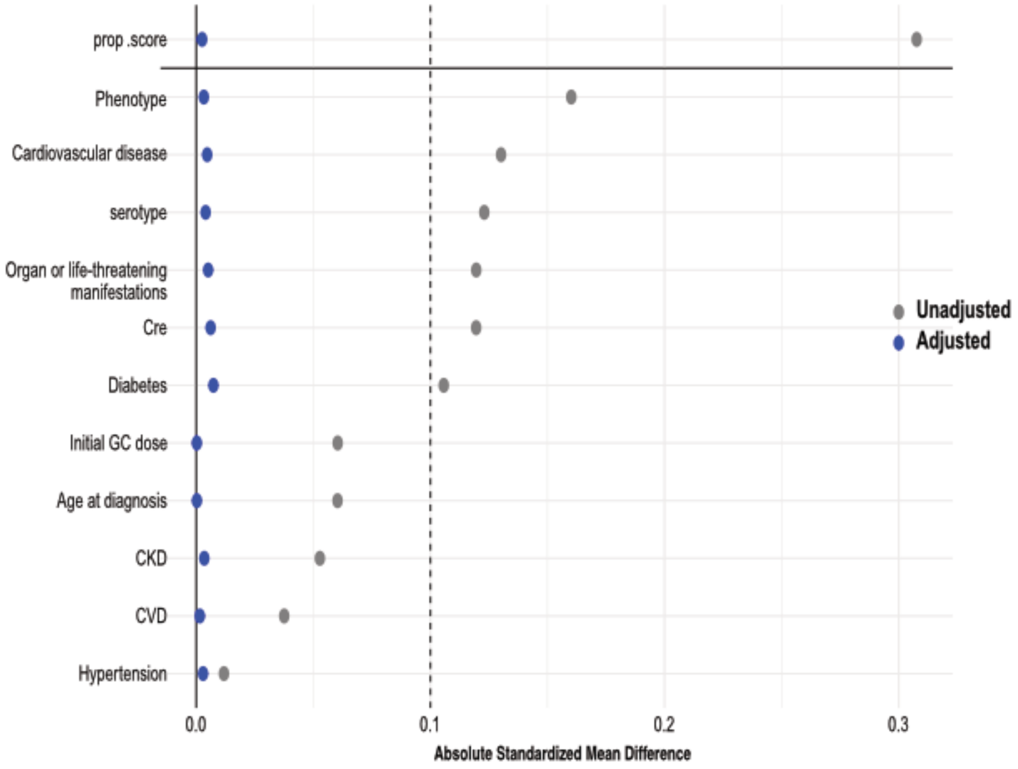

Love plot displaying the absolute standardized mean differences (SMDs) for baseline covariates between the RTX and IVCY treatment groups, before (grey points) and after (blue points) inverse probability of treatment weighting (IPW). The vertical line represents the balance threshold of SMD = 0.1. Improved covariate balance after weighting is indicated by the SMDs for most variables (blue points) falling below this threshold. Results are based on the first imputed dataset.

Abbreviations: CKD, Chronic Kidney Disease; Cre, Serum Creatinine; CVD, Cardiovascular Disease; GC, Glucocorticoid

Supplementary Fig. 5 BIC Plot for Determining the Optimal Number of Clusters

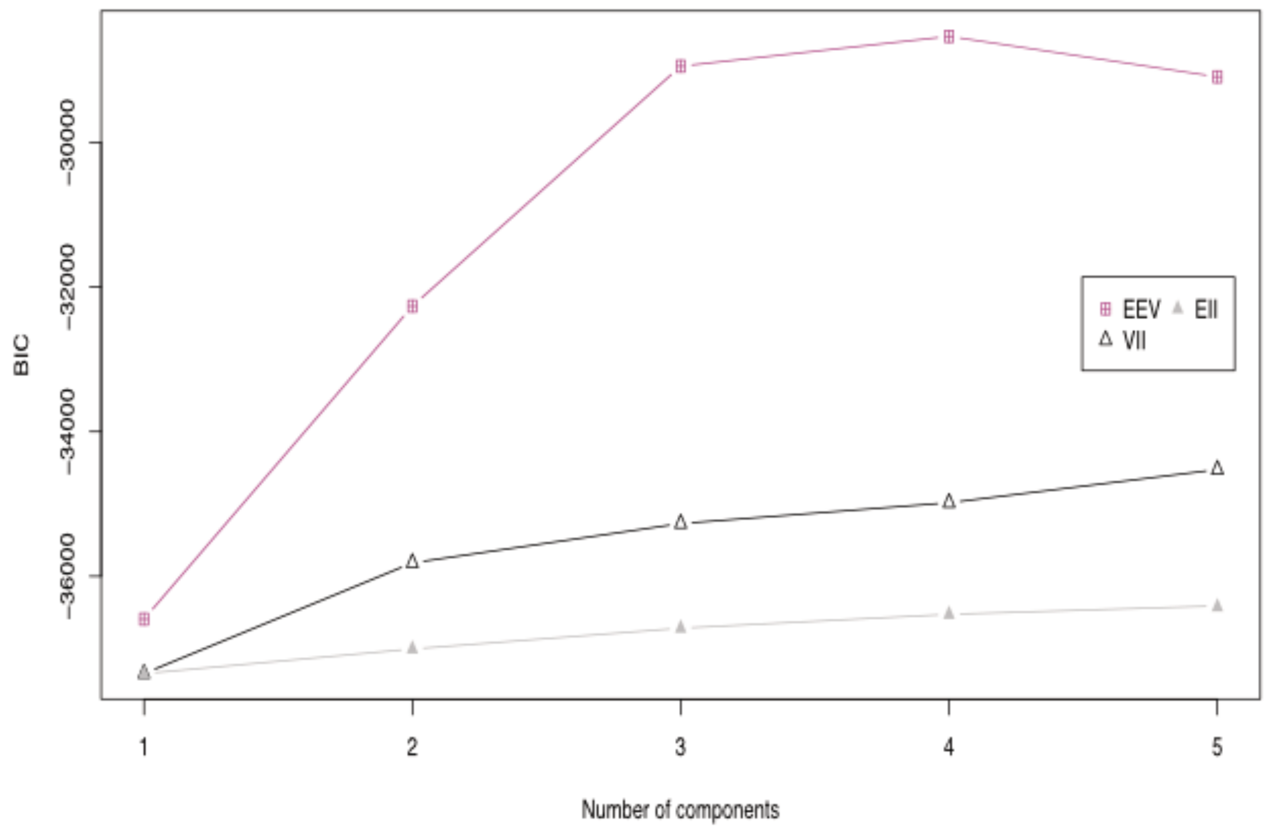

This plot displays Bayesian Information Criterion (BIC) values for Gaussian Mixture Models (GMMs) fitted with varying numbers of components (clusters) based on one representative imputed dataset (Imputation #4). For improved clarity, only the BIC curves for three selected model types, representing different covariance structures (EEV: ellipsoidal, equal volume and shape; VII: spherical, unequal volume; EII: spherical, equal volume), are shown. The highest BIC value (least negative) was achieved with the EEV model specification when assuming 4 components, suggesting that four clusters provide the optimal model fit according to the BIC criterion in this dataset.

Abbreviations: BIC, Bayesian Information Criterion; GMM, Gaussian Mixture Model

Supplementary Fig. 6 Visualization of Identified Clusters using mclustDR

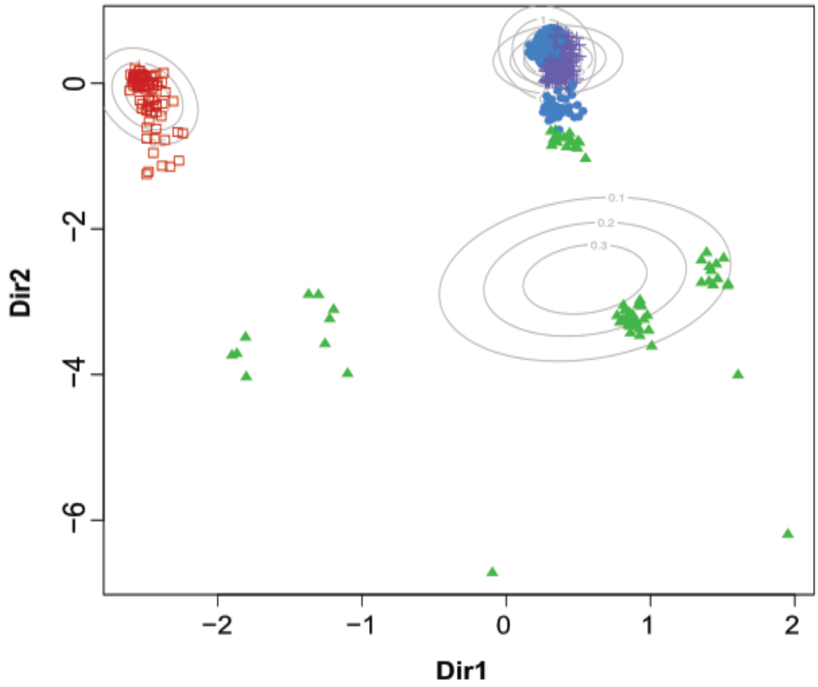

The four identified patient clusters are plotted based on the first two dimensions (Dir1 vs Dir2) derived using mclustDR from one representative imputed dataset. Points represent individual patients, colored and shaped according to their assigned cluster membership: Blue symbols = Cluster 1, Red squares = Cluster 2, Green triangles = Cluster 3, and Purple symbols = Cluster 4. Gray lines show the estimated density contours for each cluster from the fitted Gaussian Mixture Model.

Supplementary Fig. 7 Survival and Relapse Outcomes for the Four Data-Driven Clusters

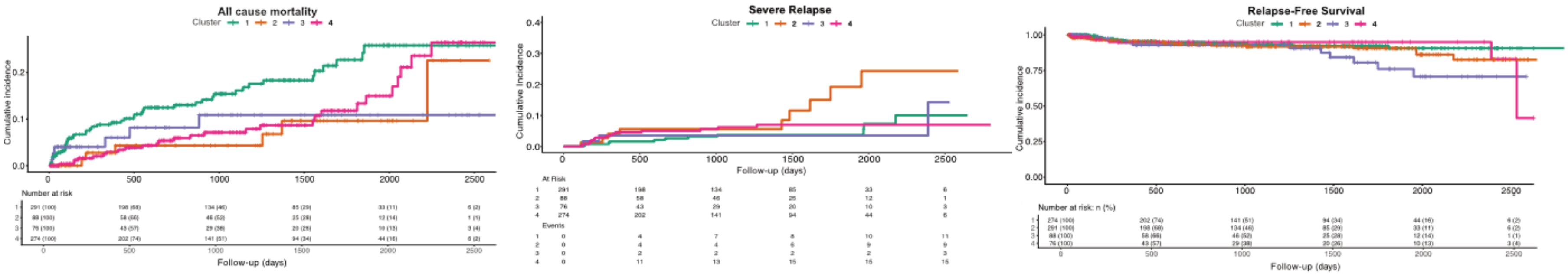

This Figure compares clinical outcomes across the four patient clusters identified in the primary data-driven analysis. The left panel shows the cumulative incidence of all-cause mortality, estimated as the complement of the Kaplan-Meier survival estimate (1 - Survival Probability). The center panel shows the cumulative incidence of severe relapse. The right panel displays Kaplan-Meier curves for relapse-free survival. Each plot compares Cluster 1 through Cluster 4, and includes tables showing the number of patients at risk over the follow-up period in days.

Supplementary Fig. 8 Concordance between Data-Driven Clusters and Conventional Classifications in the Primary Analysis

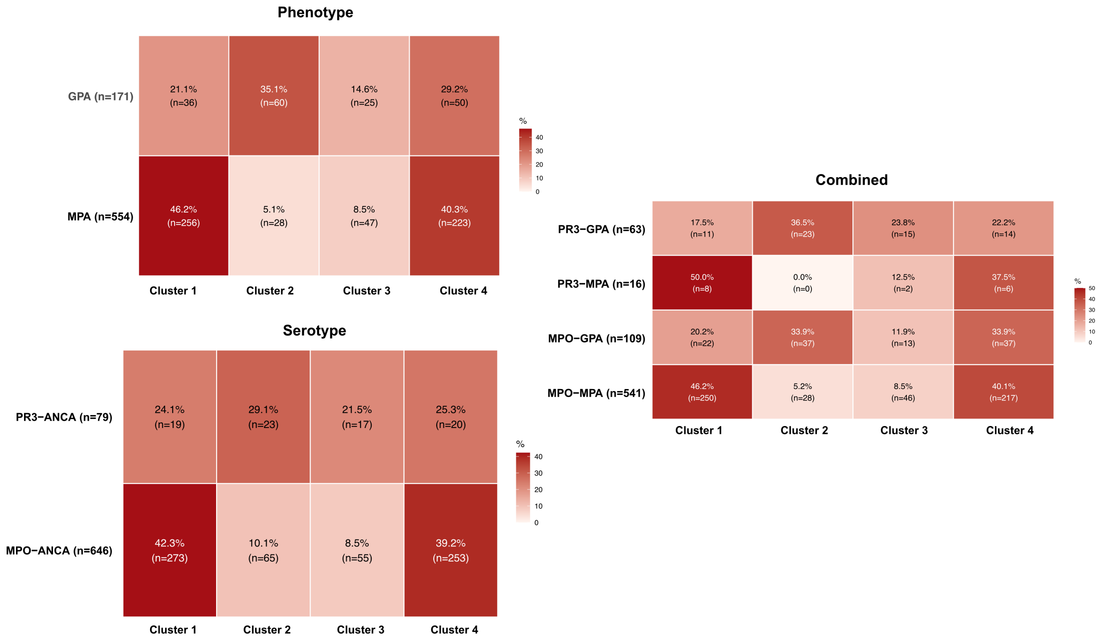

These heatmaps illustrate the concordance between conventional AAV classifications (Phenotype [top left panel], ANCA Serotype [bottom left panel], and Combined Clinico-Serological Classification [right panel]) and the four data-driven clusters identified in the primary analysis. Rows represent the conventional classification groups (displaying

total number of patients, n, for each), columns represent the four data-driven clusters, and cells indicate the percentage (reflected by color intensity) and absolute number (n) of patients from each conventional group assigned to each data-driven clusters.

Abbreviations: AAV, ANCA-Associated Vasculitis; ANCA, Anti-Neutrophil Cytoplasmic Antibody; GPA, Granulomatosis with Polyangiitis; MPA, Microscopic Polyangiitis; MPO, Myeloperoxidase; PR3, Proteinase 3.

Supplementary Fig. 9 Relapse-Free Survival: Sensitivity Analysis Including Relapsed Patients.

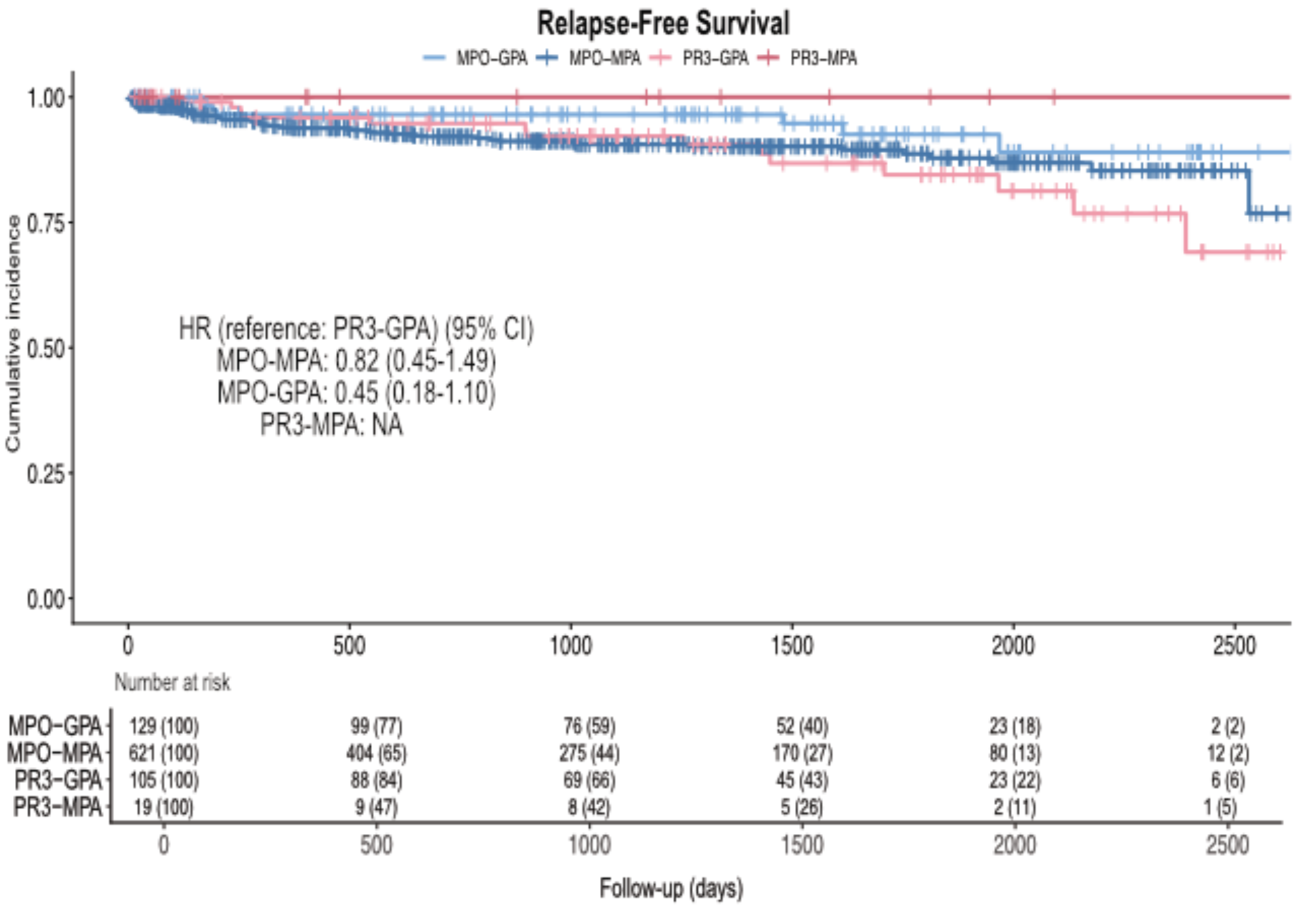

This Figure shows Kaplan-Meier curves for Relapse-Free Survival. Curves are plotted for each of the four subgroups defined by ANCA serotype (MPO/PR3) and disease phenotype (MPA/GPA): MPO-GPA, MPO-MPA, PR3-GPA, and PR3-MPA.

The table below the plot indicates the number of patients remaining at risk (i.e., relapse-free and under observation) in each subgroup at specific time points. Additionally, Hazard Ratios (HR) and their 95% Confidence Intervals (CI) for relapse are shown within the plot, comparing the MPO-MPA, MPO-GPA, and PR3-MPA groups relative to the PR3-GPA reference group.

Supplementary Fig. 10 Radar Chart for Clustering Sensitivity Analysis Including Serotype

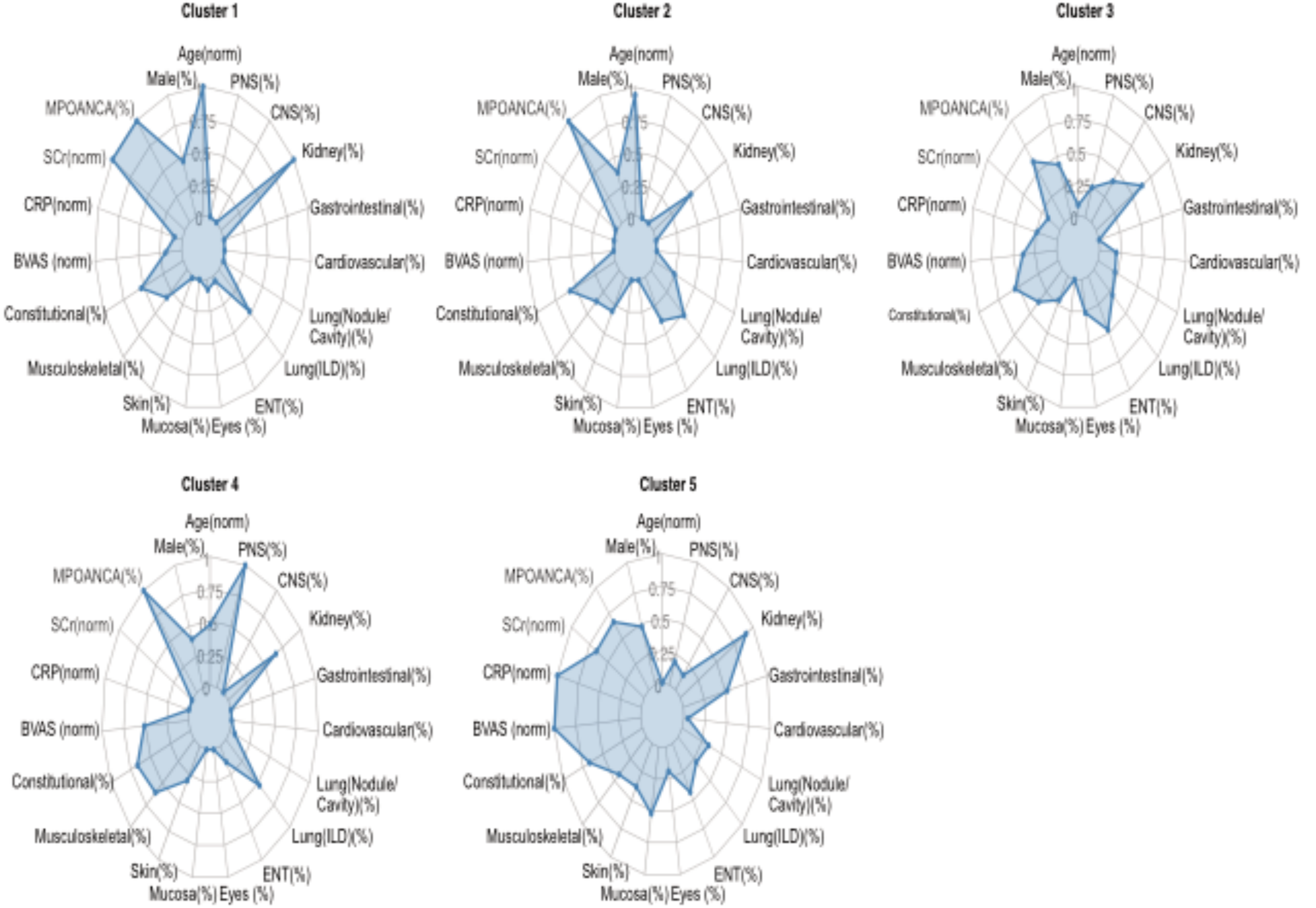
Cluster 1
Cluster 2
Cluster 3
Cluster 4
Cluster 5
Age(norm)
Male(%)
PNS(%)
CNS(%)
Kidney(%)
Gastrointestinal(%)
Cardiovascular(%)
Lung(Nodule/
Cavity)(%)
Lung(ILD)(%)
ENT(%)
Eyes (%)
Mucosa(%)
Skin(%)
Musculoskeletal(%)
Constitutional(%)
BVAS (norm)
CRP(norm)
SCr(norm)
MPOANCA(%)
0
0.25
0.5
0.75
1

This Figure presents the clinical profiles of the five distinct patient clusters identified through a sensitivity analysis where data-driven clustering (Gaussian Mixture Model) was performed *with* ANCA serotype included as an input variable. Notably, this analysis yielded five clusters, differing from the four clusters found in the primary analysis which excluded serotype.

Each radar chart corresponds to one of the five clusters. The value plotted on each axis is guided by the background grid, which uses a proportion scale (0.0 at the centre to 1.0 at the edge). The specific unit for each axis is defined by its label:

- Axes labelled with (%): These represent categorical variables. The value indicates the percentage of patients within that cluster who have the feature.
- Axes labelled with (norm): These represent continuous variables. The value indicates the cluster's mean value, normalised across all five clusters (where 0.0 is the minimum mean and 1.0 is the maximum mean)."

Abbreviations: ANCA, Anti-Neutrophil Cytoplasmic Antibody; BVAS, Birmingham Vasculitis Activity Score; CNS, Central Nervous System; CRP, C-Reactive Protein; ENT, Ear, Nose, and throat; GMM, Gaussian Mixture Model; ILD, Interstitial Lung Disease; MPO-ANCA, Myeloperoxidase Anti-Neutrophil Cytoplasmic Antibody; PNS, Peripheral Nervous System; Scr, Serum creatinine.

Supplementary Fig. 11 Sensitivity Analysis: Concordance between Data-Driven Clusters and Conventional Classifications Including Serotype

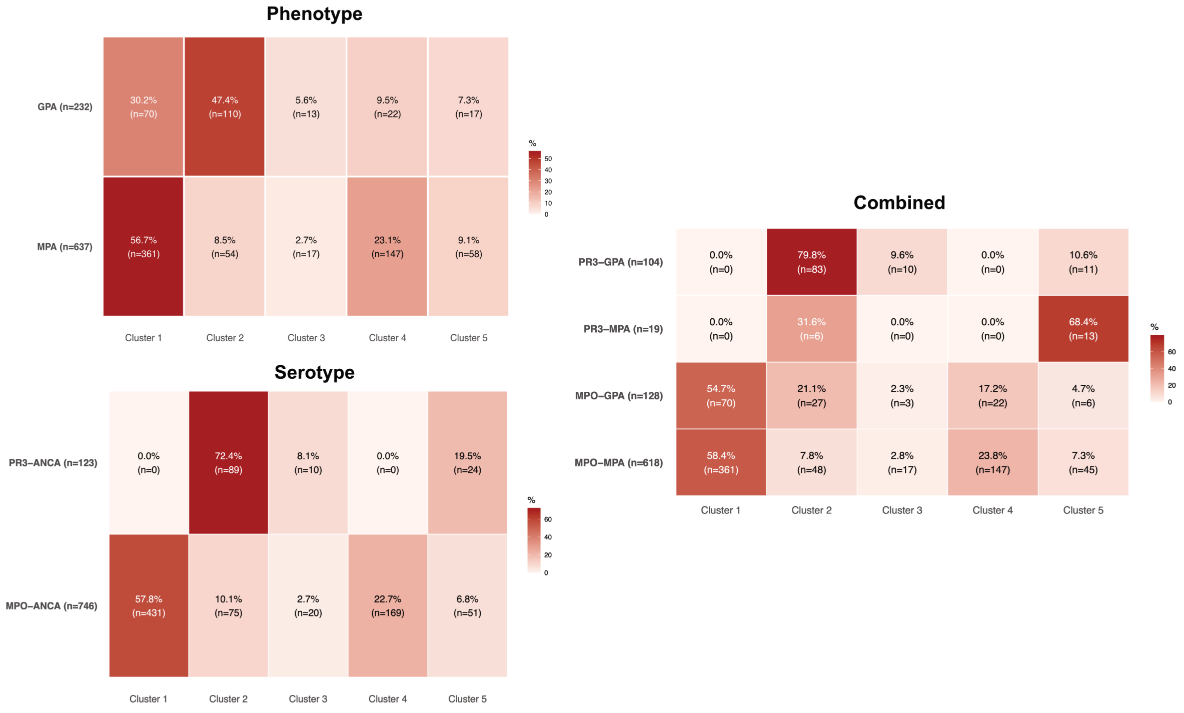

These heatmaps illustrate the concordance between traditional ANCA-associated vasculitis classifications (Phenotype [top panel], Serotype [bottom-left], and Combined sero-phenotype [bottom-right]) and the five data-driven clusters identified via Gaussian Mixture Model clustering in the sensitivity analysis that included ANCA serotype as an input variable. Rows represent the traditional groups (n=total patients), columns represent the five data-driven clusters, and cells indicate the percentage (reflected by color intensity) and absolute number (n) of patients from each traditional group assigned to each cluster.

Abbreviations: AAV, ANCA-Associated Vasculitis; ANCA, Anti-Neutrophil Cytoplasmic Antibody; GPA, Granulomatosis with polyangiitis; MPA, Microscopic polyangiitis; MPO-ANCA, Myeloperoxidase Anti-Neutrophil Cytoplasmic Antibody; PR3-ANCA, Proteinase 3 Anti-Neutrophil Cytoplasmic Antibody.
